# Conserved and diverged embryonic expression patterns of panarthropod NK-Cluster genes and new evidence for CRE-shuffling

**DOI:** 10.1186/s12862-026-02513-z

**Published:** 2026-04-03

**Authors:** Ralf Janssen, Lucia Isla Cabello, Madeleine E. Aase-Remedios

**Affiliations:** 1 Department of Earth Sciences, Palaeobiology, Uppsala University, Villavägen 16, Uppsala, 75236 Sweden; 2 Department of Biosciences, Durham University, Durham, DH1 3LE UK; 3 Marine Biology Section, Department of Biology, University of Copenhagen, Copenhagen, Denmark

**Keywords:** Arthropod evolution, Arthropod development, Homeodomain genes, NK-cluster, Cis regulatory element

## Abstract

**Background:**

The interest in the NK genes, a group of conserved homeodomain encoding transcription factors, has increased significantly in the recent years, especially among arthropods and their closest relatives. One reason is that these genes have important and (often) conserved functions in development. Another reason is that some NK genes are clustered within the genomes of different species of arthropods and indeed animals in general. The fact that this clustering has been retained for hundreds of millions of years strongly implies a biological function, and it is believed that shared cis-regulatory elements (CREs) could act to constrain the clusters. Our knowledge about the expression and function of clustered NK genes, however, is still restricted to only a few well-investigated members of this group of genes, while others have only been studied in few model species, or have been completely neglected. Recently, genomic data have provided exciting new insights into the content and clustering of NK genes in non-model species, but corresponding gene expression data are still incomplete or lacking altogether.

**Results:**

Here we present a comprehensive overview of the complement and embryonic expression pattern of NK genes in a variety of arthropod species and an onychophoran in order to gain further insight into conserved and divergent patterns of NK gene expression which we use to extrapolate the putative ancestral function(s) of these genes in panarthropods.

**Conclusions:**

We report unexpected instances of expression-pattern swapping among closely-clustered NK genes that may result from CRE-shuffling. We also discuss and conclude on the clustering and order of NK genes in the last common ancestor of bilaterian animals. Finally, we come to the conclusion that NK genes are not just involved in mesoderm evolution but are found to have conserved functions in all germ layers.

**Supplementary Information:**

The online version contains supplementary material available at 10.1186/s12862-026-02513-z.

## Background

**Fig. 1:**
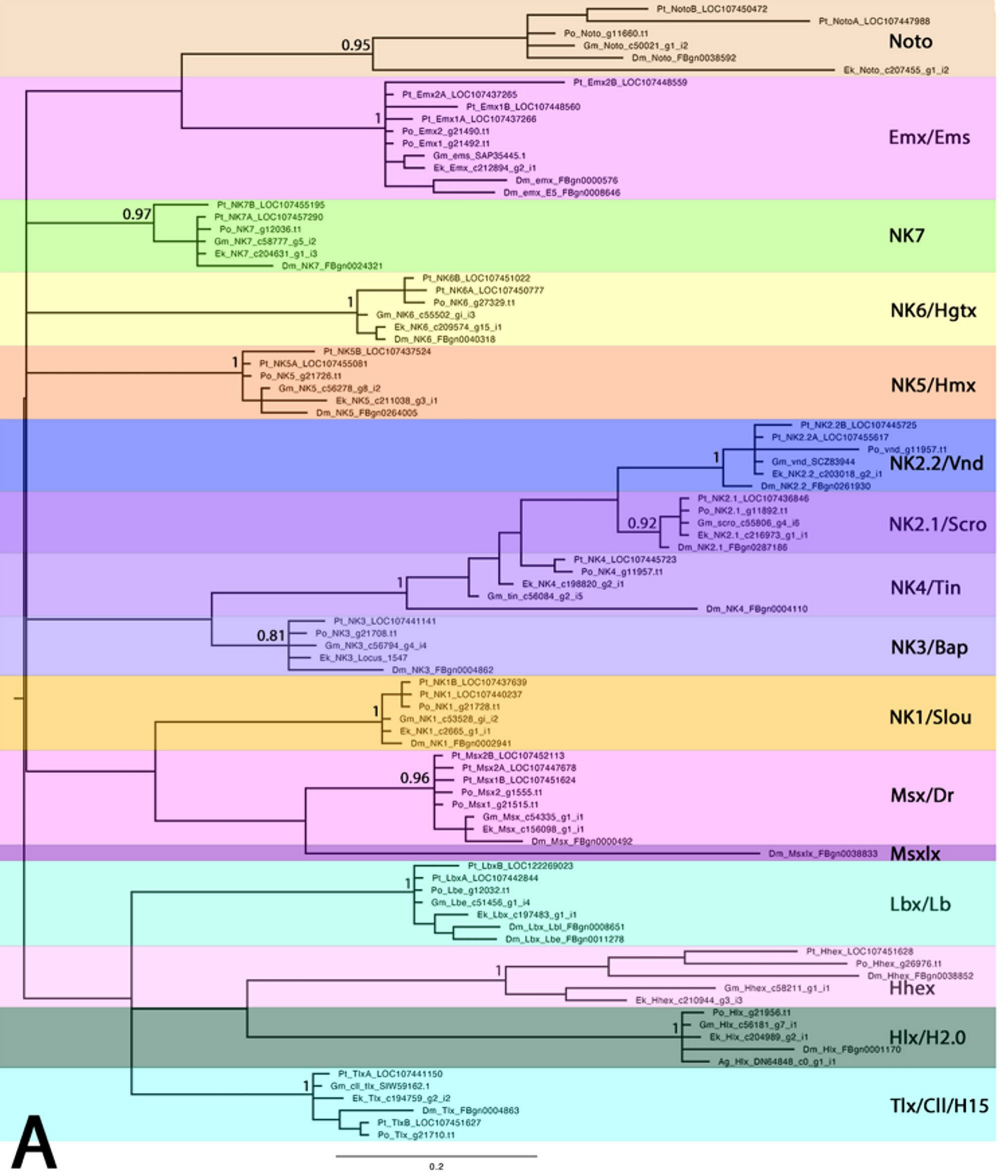

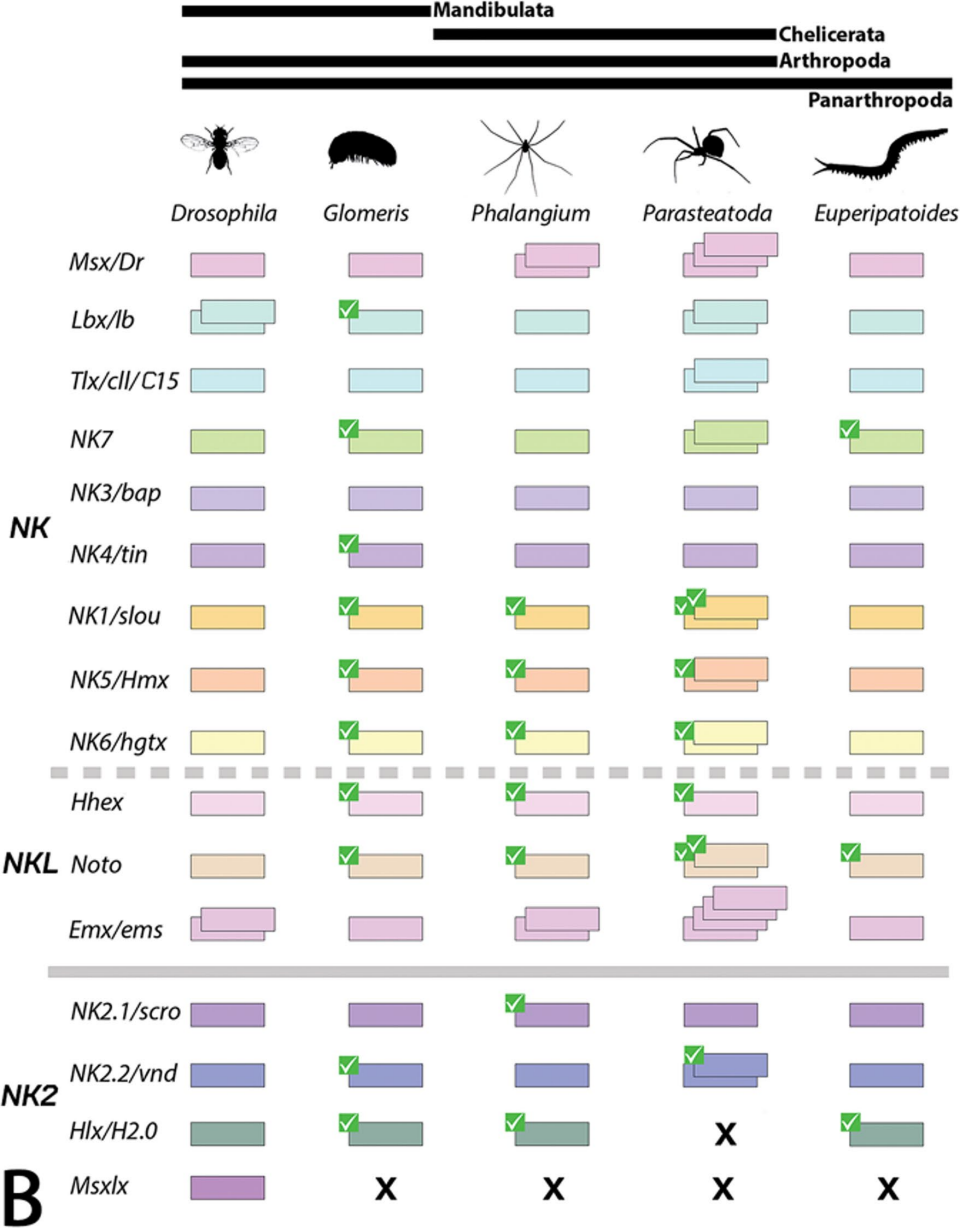
Phylogenetic analysis of NK genes and complement of NK genes in the fly *Drosophila* and in the investigated species. **A** Phylogenetic tree of NK genes. Bayesian analysis using MrBayes applying two million cycles for the Metropolis-Coupled Markov Chain Monte Carlo (MCMCMC). The tree is midpoint rooted. Node labels represent posterior possibilities. The scale bar represents 0.2 amino acid substitutions per site. Different classes of NK genes are colour-coded. Species abbreviations: Ag, *Acanthoscurria geniculata* (Chelicerata); Dm, *Drosophila melanogaster* (Insecta); Ek, *Euperipatoides kanangrensis* (Onychophora); Gm, *Glomeris marginata* (Myriapoda); Pt, *Parasteatoda tepidariorum* (Chelicerata); Po, *Phalangium opilio* (Chelicerata). **B** Complement of NK genes. Every box represents one gene (paralog). Different orthologs are colour-coded. Green check marks indicate genes for which new in-situ hybridization data have been provided in this paper. The “X” symbol stands for putative absence of the gene. Schematic drawings of the species are taken from PhyloPic (https://www.phylopic.org)

Homeobox genes are transcription factors with a characteristic DNA-binding motif, the homeodomain (HD), which evolved early in eukaryotes from related genes found in prokaryotes. Since then, the HD remained as a highly-conserved motif that is found in all groups of animals, but also fungi, protists, and plants (reviewed in [1] Ferrier 2016). In animals, there are eleven classes of homeodomain-containing genes, the CERS-class, the PRD-class, the LIM-class, the HNF-class, the SINE-class, the POU-class, the TALE-class, the PROS-class, the ZF-class, the CUT-class, and the Antennapedia (ANTP)-class ([2] Holland et al. 2007, [1] Ferrier 2016). The ANTP-class genes comprise, among others, the well-known Hox genes, the ParaHox genes and the NK genes (e.g. [2] Holland et al. 2007). Like the Hox and ParaHox genes, NK genes (named after *N*irenberg and *K*im who first discovered and investigated members of this class of genes in the vinegar fly *Drosophila melanogaster* ([3] Kim and Nirenberg 1989)) evolved by tandem duplication and are organized in clusters in bilaterian animals ([1] Ferrier 2016). In contrast to the conserved Hox cluster, however, the NK clustering is less well-conserved, and thus there are still questions and debate about the evolution and genomic organization of NK genes among animals, including arthropods (e.g. [4] Holland 2001, [5] Larroux et al. 2007, [6] Chan et al. 2015). In previous studies, the NK genes were subdivided into three groups based on their genomic organization and sequence similarity. The nine NK-cluster (NK) genes (*NK1/slou*, *NK3/bap*, *NK4/tin*, *NK5/Hmx*, *NK6/Hgtx*, *NK7*, *Msx/Dr*, *Lbx/lb* and *Tlx/cll/C15*), a number of NK-linked (NKL) genes (*Emx/ems*, *Noto*, and *Hhex*), and the NK2-cluster (NK2) genes (*NK2.1/scro*, *NK2.2/vnd*, *Hlx/H2.0* and *Msxlx*) (Fig. 1) ([1] Ferrier 2016, [7] Treffkorn et al. 2018, [8] Li et al. 2020, [9] Aase-Remedios et al. 2023, [10] Kulkarni et al. 2024) (see Table 1 for full gene names).

**Table 1:**
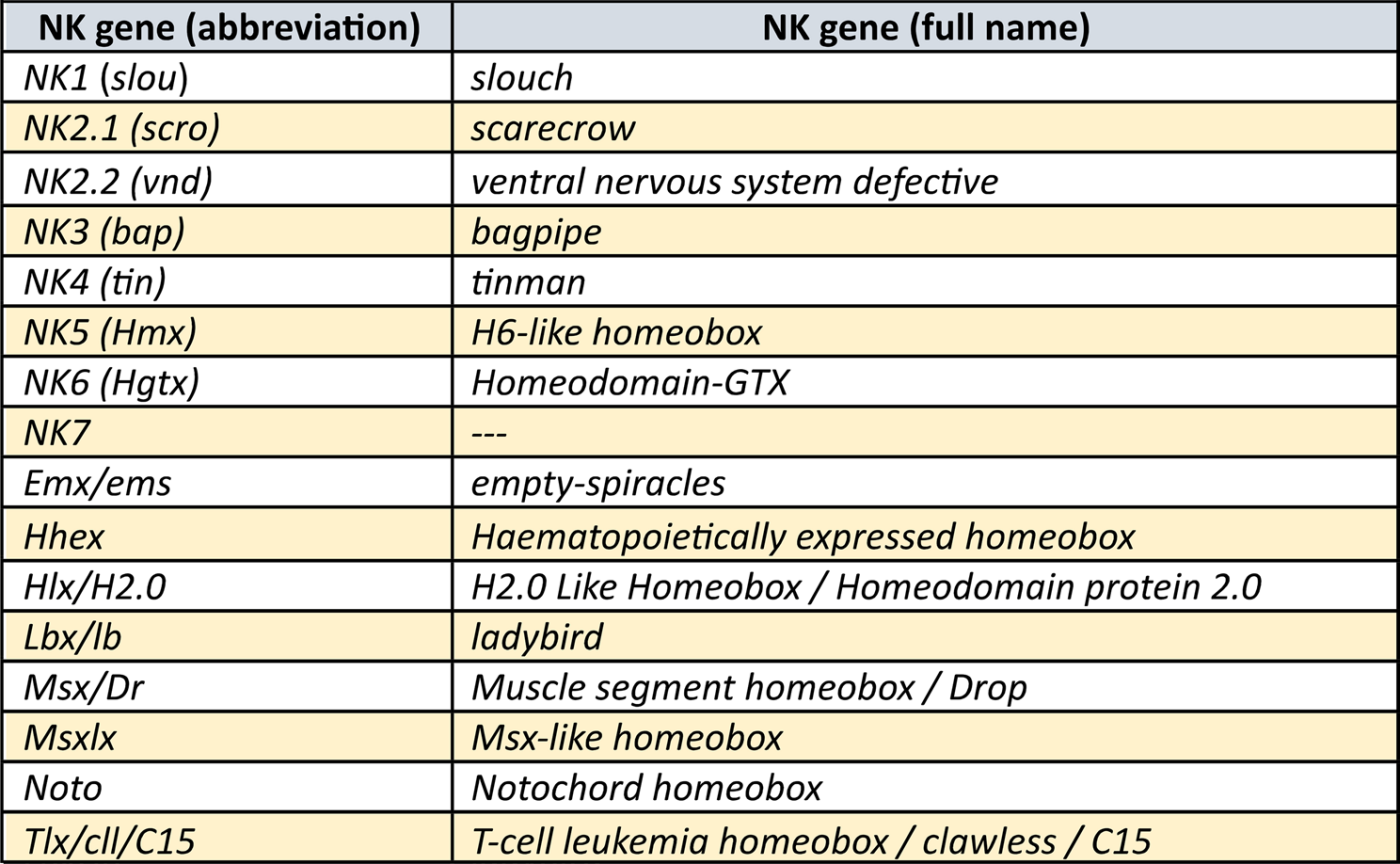
Full names of investigated NK genes

Recent studies analysing the genomes of spiders and other chelicerates revealed further insights into the clustering of NK genes, showing that in most of these species the NK cluster had largely become dispersed, though, some remnants of clustering were conserved on a two- or three-gene basis. In the study species, the same pairs or triples of genes were consistently found clustered, though lineage-specific rearrangements occurred between these gene pairs and triples, changing the larger-scale organisation of the NK cluster ([9] Aase-Remedios et al. 2023, [11] Aase-Remedios et al. 2025, [10] Kulkarni et al. 2024, [12] Klementz et al. 2025). More importantly, this work showed also that the NKL genes *Hhex*, *Noto*, and *Emx/ems* are often physically linked with the NK genes in these groups of arthropods. Since this pattern is also recognizable in the other group of protostomian animals, the spiralians/lophotrochozoans ([8] Li et al. 2020), it is likely the result of functional constraint rather than representing cases of evolutionary inertia after gene duplication.

While among the ANTP-class homeobox genes, the Hox and ParaHox genes have been studied intensively and in a plethora of species including all major groups of arthropods and their closest relatives, the onychophorans (velvet worms) and tardigrades (water bears) ([13] Weiss et al. 1998, [14] Hughes and Kaufman 2002, [15] Janssen et al. 2014, [16] Janssen et al. 2015, [17] Smith et al. 2016), the NK genes generally received much less attention, especially with respect to NKL genes that were not considered part of the core NK cluster, such as *Hhex* and *Noto*. Generally, among the published work on panarthropod NK genes, most studies address the content and clustering of NK genes (e.g [18] Ranz et al. 2022, [19] Mulhair et al. 2023, [10] Kulkarni et al. 2024, [11] Aase-Remedios et al. 2025, [12] Klementz et al. 2025), but functional data and developmental gene expression data are scarce with respect to comprehensive studies comparing and contrasting between panarthropod species. Since even in the model arthropod species *Drosophila melanogaster*, some of these genes have not (or only superficially) been studied, there is still not a single complete and comprehensive data set with respect to embryonic gene expression of these genes in any arthropod species. This impedes our understanding of the relationship between the conserved clustering and rearrangements among these genes and their functions in development.

In this study, we focus on the orthologs of the 16 previously identified clustered arthropod NK genes (NK, NK2 and NKL genes) (as defined in [9] Aase-Remedios et al. 2023 and [10] Kulkarni et al. 2024) in a myriapod, the common pill millipede *Glomeris marginata*, the common house spider *Parasteatoda tepidariorum*, the harvestman *Phalangium opilio*, and the onychophoran *Euperipatoides kanangrensis* (complementing earlier published data of NK gene expression in the closely related species *Euperipatoides rowelli* ([7] Treffkorn et al. 2018, [20] Treffkorn and Mayer 2019)) (all species mentioned in this paper are summarized in Table 2). We provide the first comprehensive overview of the content and embryonic expression patterns of NK genes in panarthropods surveying the genes and species not addressed in the patchwork of previous studies, and also discuss new genomic data on NK gene synteny in phylogenetically important branches of Ecdysozoa.

**Table 2:**
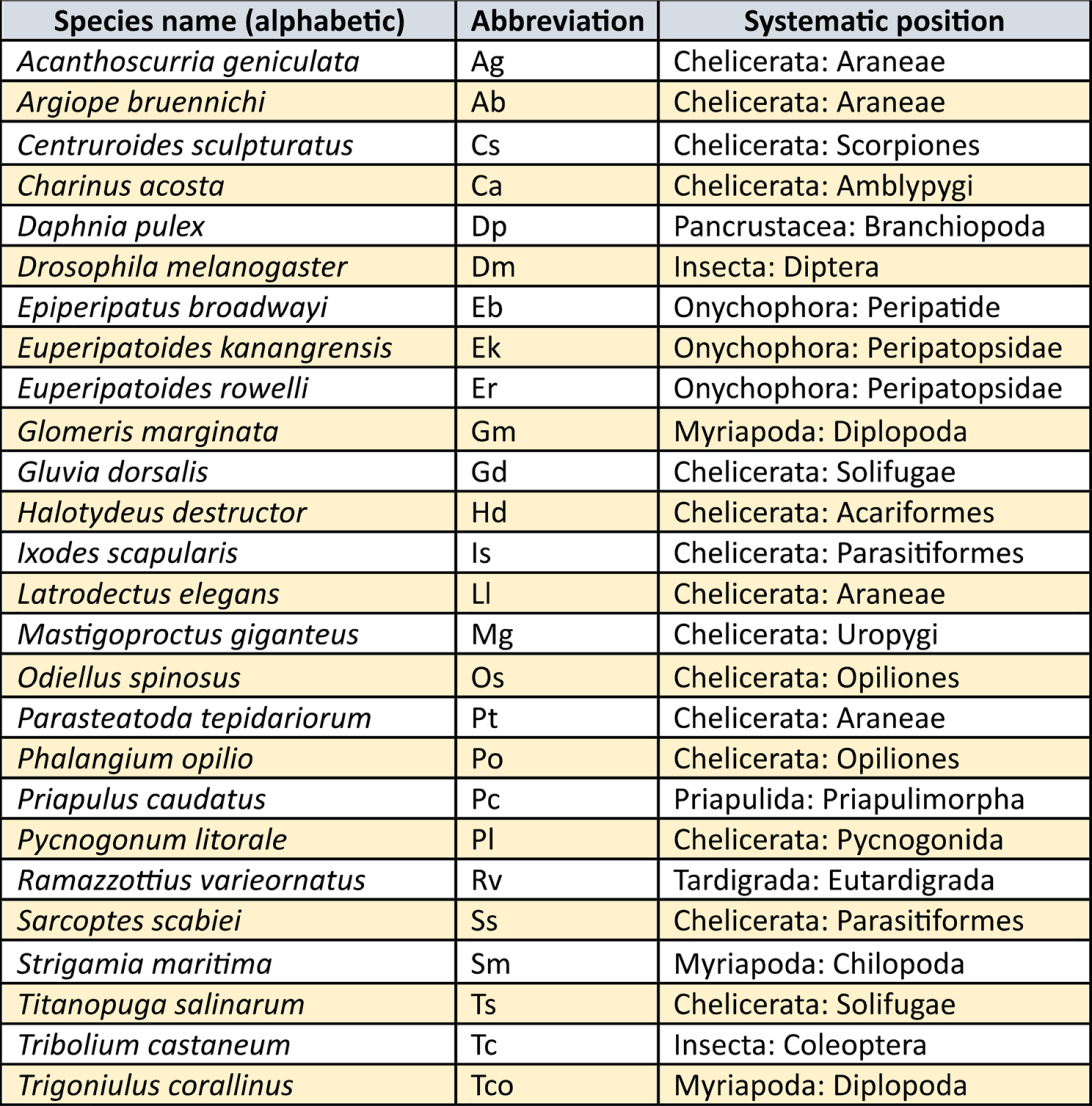
Summary of all species mentioned in this paper

With our broad-reaching comparative dataset of gene expression among different panarthropod groups, we aim to infer the ancestral expression patterns and functions of these genes in panarthropods. Generally, we find broadly conserved gene expression patterns for most NK genes among panarthropods, but also identify instances of lineage-specific changes. The latter may partially be explained by cis-regulatory element (CRE)-shuffling, i.e. a change to the target gene of a given CRE as the result of gene inversion. Finally, we find that clustered NK genes have conserved roles in the development of all three germ layers, the ectoderm, the mesoderm, and the endoderm, opposing the hypothesis that NK genes were specifically involved in mesoderm evolution and causal for the massive radiation of animal life during the Cambrian period.

## Methods

### Phylogenetic analysis of panarthropod NK genes

In order to identify NK genes from our research organisms we conducted reciprocal BLAST searches (tBLASTn) against the previously published embryonic transcriptomes of the pill millipede *Glomeris* ([21] Janssen and Posnien 2014), the harvestman *Phalangium* ([11] Aase-Remedios et al. 2025), the blue velvet worm *Euperipatoides* ([22] Janssen and Budd 2013), and the published genome of the common house spider *Parasteatoda* ([23] Schwager et al. 2017, [24] Zhu et al. 2023) using protein sequences of the candidate genes from the vinegar fly *Drosophila* as queries. The homeodomains of these genes were aligned using T-Coffee with the default parameters as provided in MacVector (version 12.6.0) (Supplementary File 1). A phylogenetic analysis was performed using MrBayes ([25] Huelsenbeck and Ronquist 2001) as previously described in [26] Panara et al. (2019). In this core analysis, two million cycles of the Metropolis-Coupled-Markov-Chain-Monte-Carlo analysis (MCMCMC) were applied. The resulting tree was midpoint rooted in FigTree (v1.4.4) and edited in Adobe Photoshop 2022 (v23.1.1). Unique sequence-identifiers are provided in the tree and the alignment (Fig. 1A, Supplementary File 1).

For extended phylogenetic analyses, we computed several additional trees. Firstly, a tree in which we added clustered and related NK genes from the priapulid worm *Priapulus caudatus* (SRX507009) to show that the investigated genes (of the core tree) are not clustering with any of the non-clustered NK genes (Supplementary Files 2 and 3); in this analysis, three million cycles MCMCMC were applied. In a second additional tree we added the sequences of the red flour beetle *Tribolium castaneum*, the centipede *Strigamia maritima*, the water flea *Daphnia pulex* and the tardigrade *Ramazzottius varieornatus* (Supplementary Files 4 and 5); in this analysis, five million cycles MCMCMC were applied. A third additional tree included the sequences of chelicerates including the tick *Ixodes scapularis*, the mite *Halotydeus desctructor*, the scorpion *Centruroides sculpturatus*, the vinegaroon *Mastigoproctus giganteus*, the pycnogonid *Pycnogonum litorale*, and the whip scorpion *Charinus acosta* (Supplementary Files 6 and 7); in this analysis, four million cycles MCMCMC were applied. A fourth additional tree summarizes the NK sequences of myriapods and *Drosophila* (Supplementary Files 8 and 9); in this analysis, two million cycles MCMCMC were applied. All trees were computed as described above.

### Animal husbandry, polymerase chain reaction (PCR), and whole-mount in situ hybridization (WISH)

Embryos were obtained and treated as described in [27] Janssen et al. (2004) (*Glomeris*), [28] Prpic et al. (2008) (*Parasteatoda*), [29] Janssen et al. (2021) (*Phalangium*), [30] Grossmann and Prpic (2012) (*Tribolium*) and [31] Janssen et al. (2010) (*Euperipatoides*). Fragments of the investigated candidate genes were amplified by means of RT-PCR using gene specific primers. cDNA was synthesized from mRNA isolated from total RNA extractions using the Dynabeads mRNA Purification Kit (Invitrogen, cat. No. 61006). Reverse primers were designed with 5´-T7 promotor overhangs for direct use of purified PCR products (QIAquick PCR purification system (QIAGEN, cat No. 28104)) as templates for RNA probe synthesis ([32] David and Wedlich 2001) (see Supplementary File 10 for information on primer sequences). Digoxigenin (DIG)-labelled antisense-RNA probes were purified using the RNeasy Mini Kit (QIAGEN, cat. No. 74104). For all species, the same universal whole mount in situ hybridizations (WISH) protocol was used [33] (Janssen et al. 2018). After WISH, SYBR-green counter-staining was applied to embryos using a 1:10000 dilution of the dye in phosphate buffered saline with 0.1% Tween-20 (PBST) for approximately 20 min. Excess dye was removed by several washing steps in PBST for at least 30 min.

### Data documentation

Bright-field microscopy and visualization of the nuclear dye SYBR-green were performed under a MZ-FLIII dissection microscope (Leica) equipped with a DC490 digital camera (Leica). Linear adjustments on contrast, colour, and brightness of taken pictures were performed with Adobe Photoshop 2022 (v23.1.1).

## Results

### NK cluster genes of the panarthropod species investigated in this paper

NK genes of the velvet worm *Euperipatoides rowelli* have previously been studied ([7] Treffkorn et al. 2018, [20] Treffkorn and Mayer 2019). We identified single copies of all NK genes previously described for *Euperipatoides rowelli* in *Euperipatoides kanangrensis*, including *NK6* (Figs. 1A, B). A closer look at the described two *NK6* paralogs in *Euperipatoides rowelli* suggests that these could represent isoforms rather than true paralogs (see [7] Treffkorn et al. 2018). In addition, we also found orthologs of *NK7*, *Noto* and *Hlx/H2.0* which have not been identified in *Euperipatoides rowelli*, though it is unclear if this reflects a loss in the latter species, or incomplete transcriptome sequencing. In both species of *Euperipatoides*,* Msxlx* was not found (Fig. 1A, B). In the spider *Parasteatoda* several NK genes have been retained as two copies (Fig. 1A, B). Beyond that, *Emx/ems* and *Msx/Dr* are present in four and three copies respectively in this species (Fig. 1A, B), while *Hlx/H2.0* and *Msxlx* could not be identified in this species (also see [9] Aase-Remedios et al. 2023). However, we identified a *Hlx/H2.0* gene in the mygalomorph spider *Acanthoscurria geniculata* (Fig. 1A). In another chelicerate, the distantly related harvestman *Phalangium*, all NK genes except *Emx/ems* and *Msx/Dr* are present as single copies (Fig. 1A, B). The harvestman possesses two paralogs of both *Emx/ems* and *Msx/Dr*. We did not identify an ortholog of *Msxlx* in the harvestman (Fig. 1A, B). In the millipede *Glomeris*, all NK genes are present as single copies except for *Msxlx* which could not be identified in this species (Fig. 1A, B).

### Embryonic expression patterns of panarthropod NK genes

**Fig. 2:**
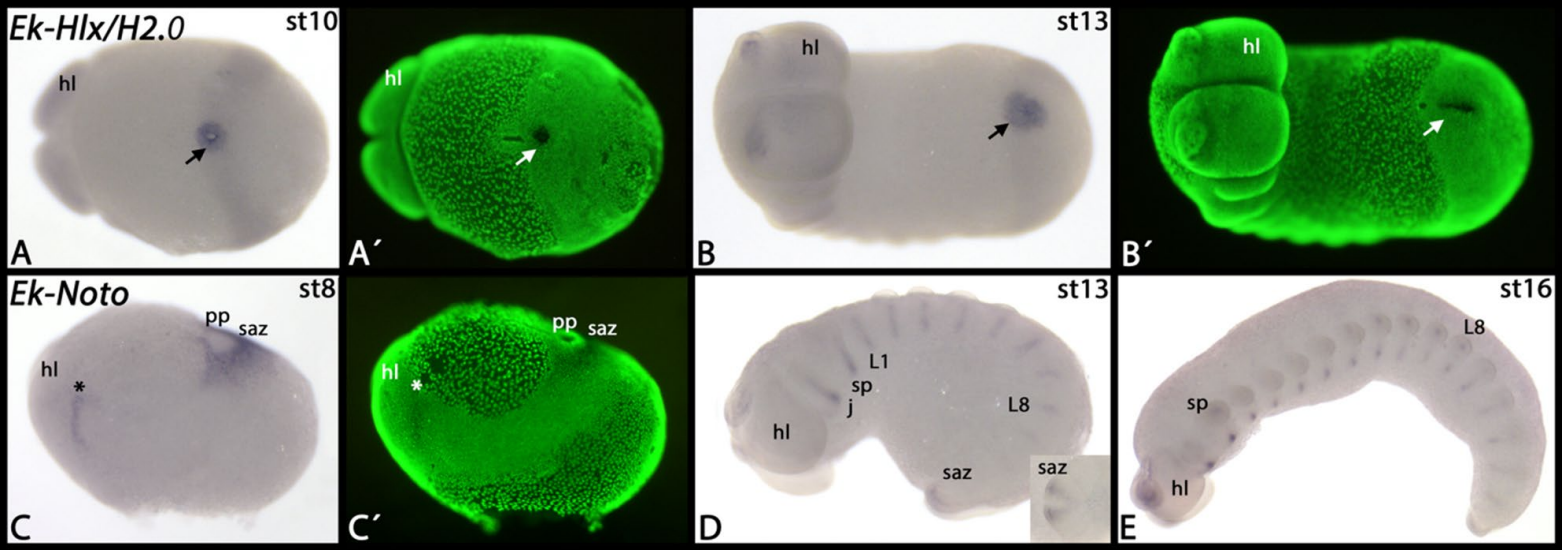
Expression of *Euperipatoides Hlx/H2.0* and *Noto*. In all panels, anterior is to the left. Panels **A** and **B** represent ventral views; panels **C**-**E** represent lateral views. Note expression of *Hlx/H2.0* in the posterior region of the embryos (arrows in **A** and **B**). The asterisk in panel **C** marks a stripe of expression at the interface between the head lobes and the jaw-bearing segment. The inset in panel **D** shows a ventral view on the posterior of the embryo. Panels marked with an apostrophe represent SYBR-green staining of embryos in corresponding panels. Developmental stages are indicated in the top right corner of each panel. Abbreviation: hl, head lobe; j, jaw-bearing segment; L, leg-bearing segment; pp, posterior pit; saz, segment addition zone; sp, slime papilla-bearing segment

Most of the NK genes have been investigated in the onychophoran *Euperipatoides rowelli* ([7] Treffkorn et al. 2018, [20] Treffkorn and Mayer 2019), but in *Euperipatoides kanangrensis* we identified three additional NK genes, *NK7*, *Noto* and *Hlx/H2.0* (Fig. 1A, B). The *Hlx/H2.0* gene is exclusively expressed in the posterior pit region that likely contributes to the early developing mesoderm (discussed in [34] Janssen and Budd 2024) (Fig. 2A, B). Faint signal is visible throughout the embryo and inside the frontal appendages. This, however, could represent background staining or artificial staining due to prolonged staining time. *Euperipatoides Noto* is first expressed in a broad field surrounding the posterior pit, but the pit itself does not express *Noto* (Fig. 2C). Additionally, there is a stripe of expression at the interface of the head lobes and the jaw-bearing segment (Fig. 2C). Later, additional stripes appear in consecutive posterior segments (Fig. 2D). The posterior expression around the posterior pit remains, but the domain shrinks at later developmental stages (Fig. 2D, inset). Again later, the stripes transform into patches of expression ventral to the appendages (Fig. 2E). This expression is likely associated with the developing ventral nervous system ([35] Whitington and Mayer 2011, [33] Janssen et al. 2018). The *NK7* gene is expressed ubiquitously (not shown).

**Fig. 3:**
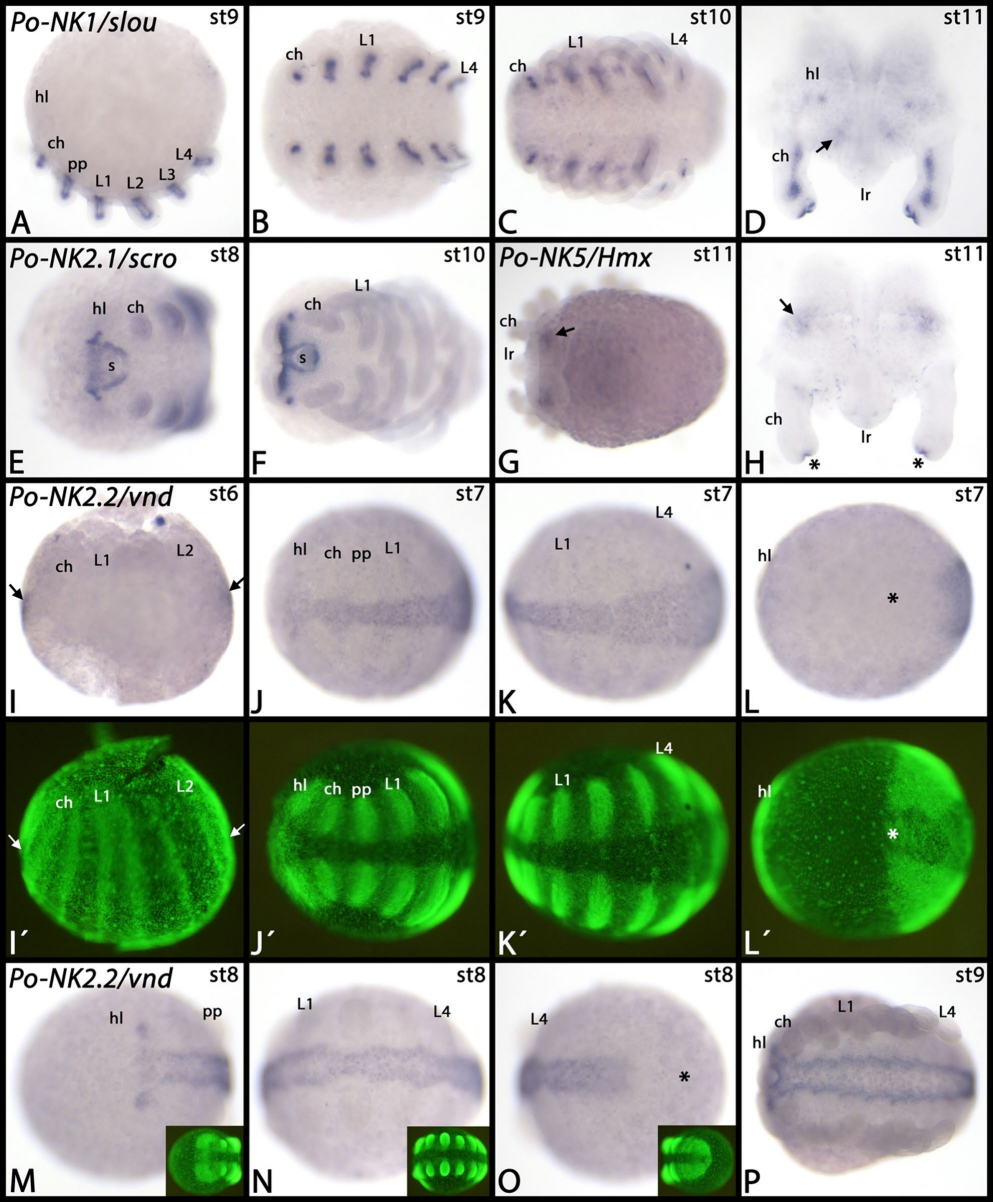
Expression of *Phalangium* NK genes *NK1/slou*, *NK2.1/scro*, and *NK2.2/vnd*. In all panels, anterior is to the left (except for panels **D** and **H** which present dissected head lobes and chelicerae; in these panels anterior is up). The arrow in panel **D** marks dot-like expression in the head lobe. The arrows in panels **G** and **H** mark broad domains of expression in the head lobe. Asterisks in panel **H** mark unspecific signal on the tips of the chelicerae that appears at late developmental stages. Arrows in panel **I** point to anterior and posterior expression. Asterisks in panels **L** (dorsal view) and **O** (posterior view) mark the posterior of the embryos. Panels marked with an apostrophe and insets in panels **M**-**O** represent SYBR-green staining of embryos in corresponding panels. Developmental stages are indicated in the top right corner of each panel. Abbreviations: ch, chelicera; hl, head lobe; L, leg; lr, labrum; pp, pedipalp; s, stomodaeum

**Fig. 4:**
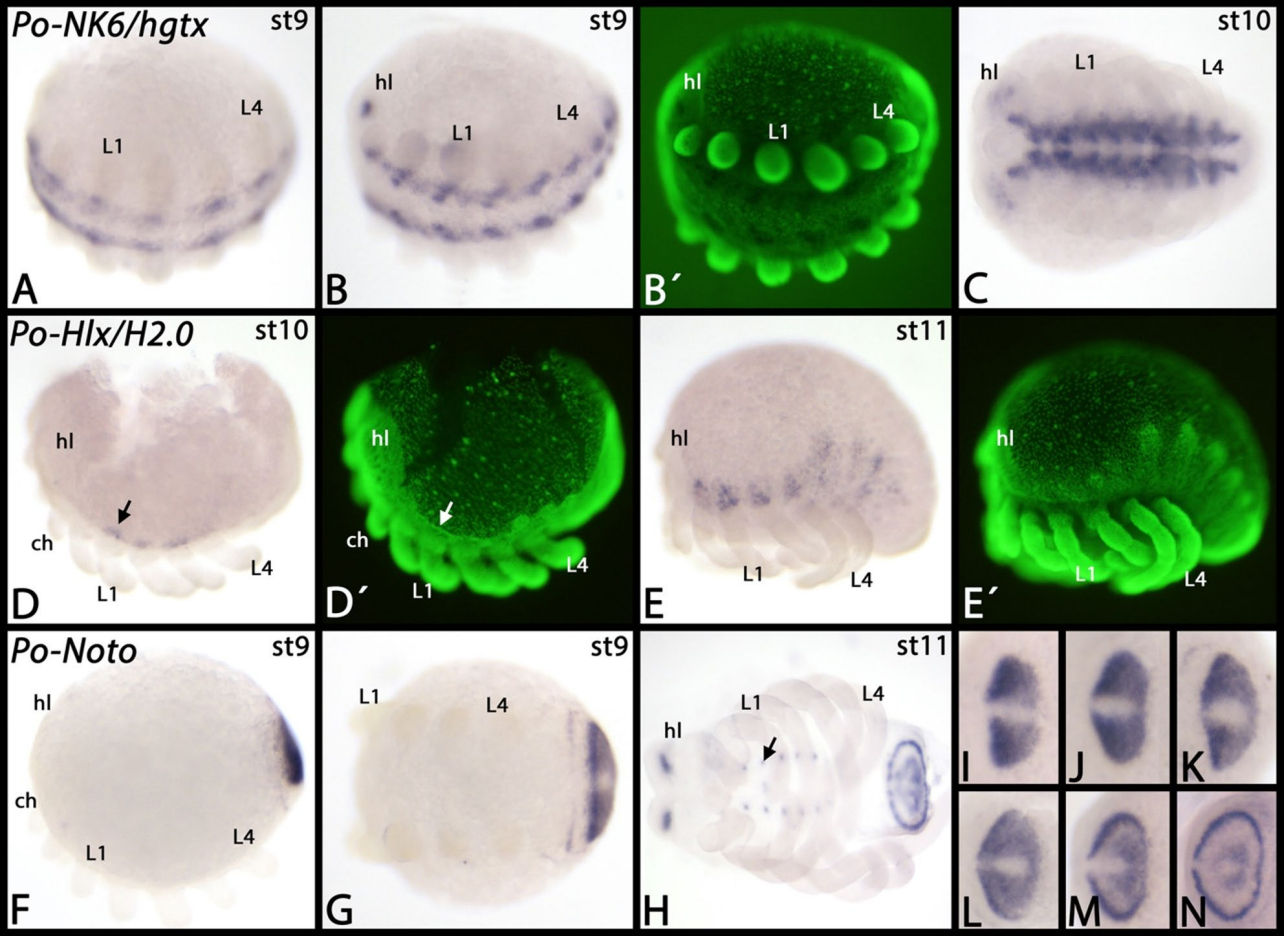
Expression of *Phalangium* NK genes *NK6/hgtx*, *Hlx/H2.0*, and *Noto*. In all panels, anterior is to the left. The arrow in panel **D** points to lateral expression. The arrow in panel **H** points to expression in the central nervous system. Panels marked with an apostrophe represent SYBR-green staining of embryos in corresponding panels. Developmental stages are indicated in the top right corner of each panel. Abbreviations: ch, chelicera; hl, head lobe; L, leg

*Phalangium NK1/slou* is expressed inside the developing appendages and this expression most likely represents mesodermal tissue (Fig. 3A-D). Within the head lobes, there are a few distinct dots of expression (Fig. 3D). *Phalangium NK2.1/scro* is expressed in a specific pattern in the anterior region of the embryo where the brain develops, and around the stomodaeum (Fig. 3E, F). *Phalangium NK5/Hmx* is exclusively expressed as two broad domains in the developing head lobes (Fig. 3G, H). *Phalangium NK2.2/vnd* is first expressed in the very anterior and the very posterior of the ventral midline (Fig. 3I). Later it is expressed in the complete ventral midline area, except for the very anterior and posterior ends of the developing embryos (Fig. 3J-P). *Phalangium NK6/hgtx* is expressed in both the developing brain and the ventral nerve cord (Fig. 4A-C). *Phalangium Hlx/H2.0* is expressed in a salt-n-pepper pattern dorsal to the base of the pedipalps and the legs (Fig. 4D, E). Although we could isolate *Phalangium Hhex* from embryonic cDNA, we were unable to detect specific expression of this gene; possibly it is expressed at stages we did not investigate, or is expressed too faintly to be detected, or is expressed ubiquitously at very low level (not shown). *Phalangium Noto* is expressed in the posterior segment addition zone (SAZ) and in newly formed posterior segments. In the SAZ, expression is dynamic, and in the newly formed segments expression is as a distinct transverse stripe that disappears quickly from the most ventral region, and slightly later also from the rest of the segment (Fig. 4F-N). At late developmental stages, additional expression appears in the region where the eyes will form (cf. [36] Gainett et al. 2024a), and in the developing ventral nervous system as few distinct dots per segment (Fig. 4H). Note the dynamic expression within the SAZ (Fig. 4I-N).

**Fig. 5:**
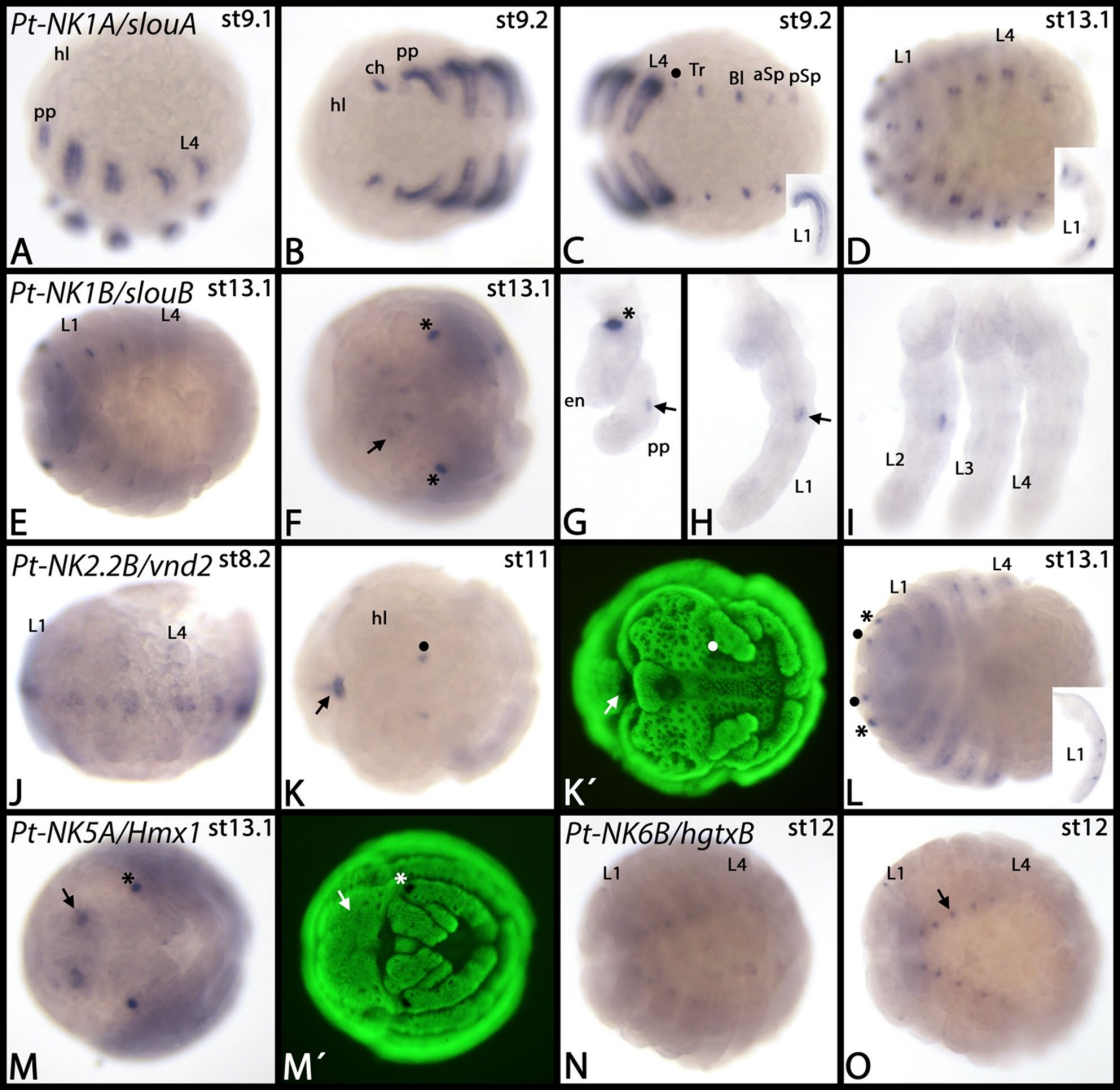
Expression of *Parasteatoda NK1A/slouA*, *NK1B/slouB*, *NK2.2B/vnd2*, *NK5A/Hmx1*, and *NK6B/hgtxB*. In all panels, anterior is to the left (except panels **G**-**I** and insets in panels **C**, **D** and **L** which show dissected appendages; in these panels dorsal is to the right). The filled circle in panel **C** marks a small domain of expression in the first opisthosomal segment. The filled circle in panel **K** marks dots of expression in the cheliceral segment. The arrow in panel **K** points to posterior expression. The arrow in panel **M** points to broad domains of expression within the head lobes. Asterisks in panels **G**, **L** and **M** mark unspecific signal in the egg teeth. The arrow in panel **O** points to dots of expression along the ventral midline of the embryo (legs have been removed from this embryo). Panels marked with an apostrophe represent SYBR-green staining of embryos in corresponding panels. Developmental stages are indicated in the top right corner of each panel. Abbreviations: aSp, anterior spinnerets; Bl, book lungs; ch, chelicera; en, endite; hl, head lobe; L, leg; pp, pedipalp; pSp, posterior spinnerets; Tr, tracheae

**Fig. 6:**
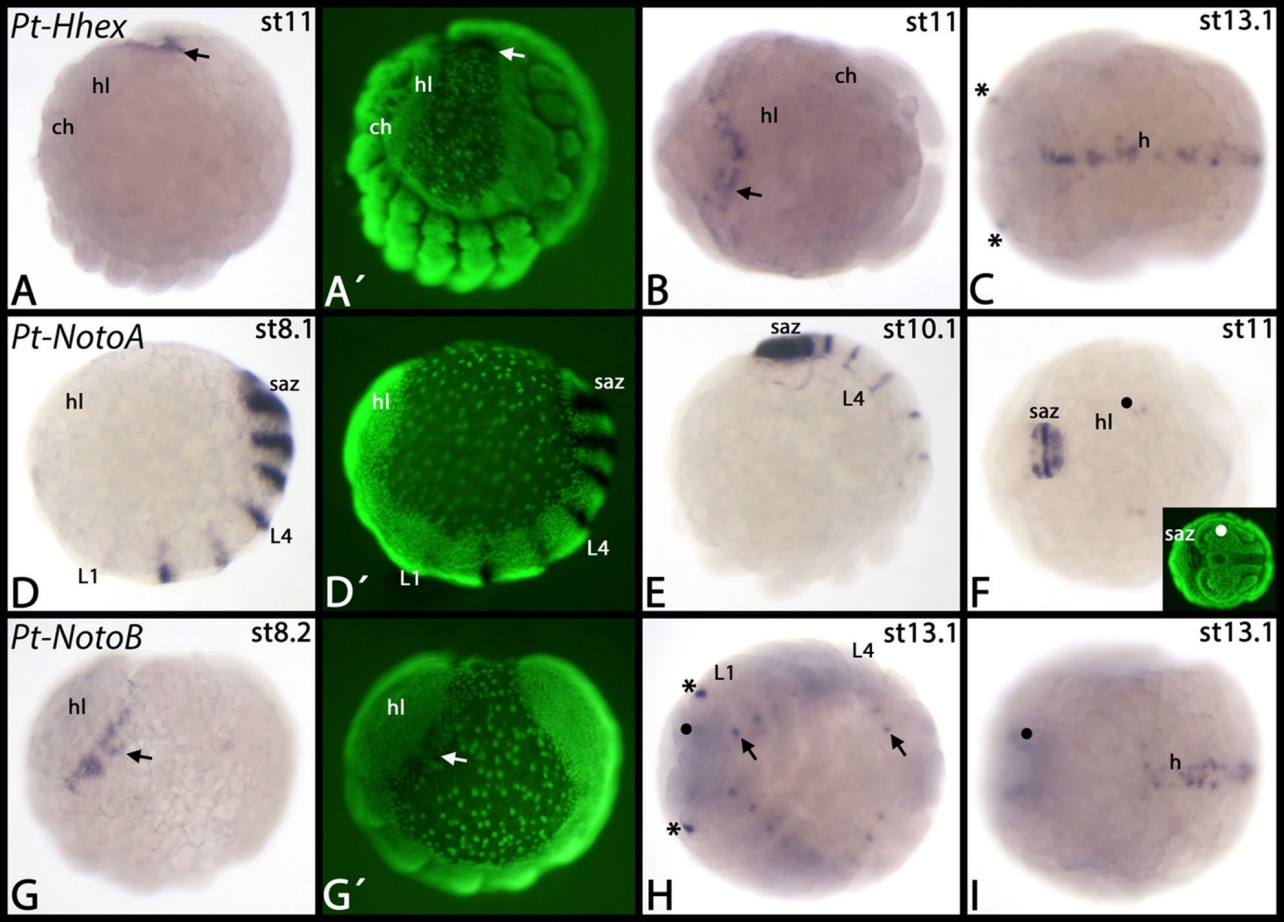
Expression of *Parasteatoda Hhex*, *NotoA*, and *NotoB*. In all panels, anterior is to the left. The arrows in panel **A** and **B** point to expression at the interface of the posterior region of the embryo and the head of the embryo. Asterisks in panels **C** and **H** mark unspecific signal in the egg teeth. The filled circle in panel **F** marks faint dots of expression within the head lobes. The arrow in panel **G** points to expression lateral to the head lobes. The arrows in panel **H** mark dot-like domains along the ventral midline. The filled circles in panels **H** and **I** mark expression within the head lobes. Panels marked with an apostrophe represent SYBR-green staining of embryos in corresponding panels. Developmental stages are indicated in the top right corner of each panel. Abbreviations: ch, chelicera; h, heart; hl, head lobe; L, leg; pp, pedipalp; saz, segment addition zone

*Parasteatoda NK1A/slouA* (called *NK-1-B-1* in [9] Aase-Remedios et al. 2023) is expressed in the mesoderm of all developing appendages including the opisthosomal spinnerets and breathing organs (Fig. 5A-D). Interestingly, there is also faint and transient expression in the first opisthosomal segment that does not develop any appendicular structures (Fig. 5C). Later during development, there are only a few (likely mesodermal) patches of expression within the developing appendages (Fig. 5D). The initial expression of the second paralog, *NK1B/slouB* (called *NK-1-B-2* in [9] Aase-Remedios et al. 2023) starts much later than that of *NK1A/slouA* and its expression is restricted to a small domain within the pedipalps and the first two pairs of legs (Fig. 5E-I). Additionally, there are some dots of expression in the head lobes (Fig. 5F). In early germ band stage embryos, *Parasteatoda NK2.2B/vnd2* is expressed along the complete ventral midline (Fig. 5J), but in later stages expression is restricted to the anterior and the very posterior of the developing embryo (Fig. 5K). The anterior expression appears to be in the posterior region of the head lobes (Fig. 5K). Later, however, additional expression appears as several dots in the appendages (Fig. 5L). It is unclear if this represents mesodermal tissue, or derivatives of the peripheral nervous system. *Parasteatoda Nk5A/Hmx1* is exclusively expressed in the head lobes and at late developmental stages (Fig. 5M). Note that we did not detect any other expression of this gene suggesting that the previously described expression represents a staining artefact (cf. [37] Leite et al. 2018, their supplementary data). Likely, the expression of the two *NK5* paralogs represents a case of temporal sub-functionalization because the second paralog is expressed in a comparable pattern, but at earlier developmental stages (cf. [37] Leite et al. 2018, their supplementary data). *Parasteatoda NK6B/hgtxB* is exclusively expressed in a subset of cells of the ventral nerve cord at relatively late stages of embryonic development (Fig. 5N, O). Similarly, the second paralog, *NK6A/hgtxA* (called *Nkx6.2* in the cited studies), is expressed in the central nervous system ([38] Leite et al. 2024, [39] Medina-Jiménez et al. 2024a). *Parasteatoda Hhex* is first expressed at approximately stage 11 at the interface between the head lobes and the SAZ which abut at this stage of development (Fig. 6A, B) (cf. [40] Mittmann and Wolff 2012). This expression is correlated with the so-called extra-embryonic tissue (reviewed in [41] Prpic and Pechmann 2022). Later during development, *Hhex* is expressed along the dorsal midline of the embryo which is likely associated with the development of the dorsal tube (the heart of the spider) (Fig. 6C) (cf. [42] Janssen and Damen 2008). *Parasteatoda NotoA* is first expressed at the germ disc stage and when the early germ band forms it is expressed as transverse stripes in the segments ([43] Akiyama-Oda and Oda 2020, [44] Oda and Akiyama-Oda 2020). Later, it is expressed in the segment addition zone and as distinct transverse stripes in newly formed posterior segments (Fig. 6D-F). As these segments mature, expression becomes weaker and finally disappears from the segments (Fig. 6D-F). Additional expression is as two weak but distinct spots in the head lobes (Fig. 6F). *Parasteatoda NotoB* is first expressed on either side of the developing head lobes in the extraembryonic tissue (Fig. 6G). Later, this expression disappears and instead expression appears as distinct dots in the developing brain, the ventral nerve cord, and the tail region of the spider embryo (Fig. 6H, I).

**Fig. 7:**
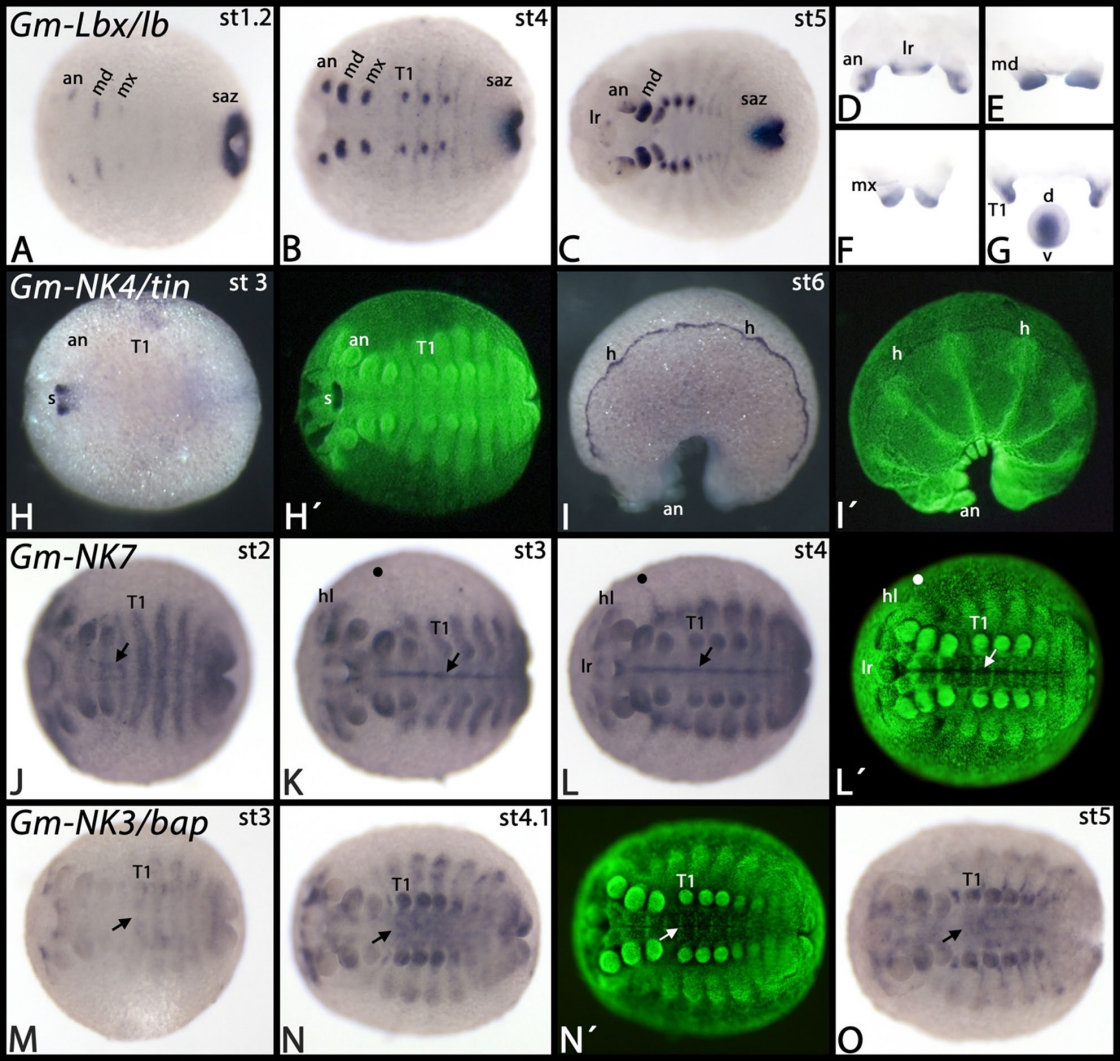
Expression of *Glomeris Lbx/lb*, *NK4/tin*, *NK7*, and *NK3*. In all panels, anterior is to the left, (except panels **D**-**G** which represent dissected appendages and anterior views). The arrows in panels **J**-**L** mark expression in the midline. The filled circles in panels **K** and **L** mark expression in the extraembryonic tissue. Arrows in panels **M**-**O** mark the anterior border of expression in the visceral mesoderm. Panels marked with an apostrophe represent SYBR-green staining of embryos in corresponding panels. Developmental stages are indicated in the top right corner of each panel. Abbreviations: an, antenna; d, dorsal; h, heart; lr, labrum; md, mandible; mx, maxilla; T, trunk segment; s, stomodaeum; saz, segment addition zone; v, ventral

*Glomeris Lbx/lb* is first expressed in the segment addition zone and the primordia of the head appendages (Fig. 7A). Later, expression also appears in the leg primordia and as faint thin stripes at the borders of the trunk segments (Fig. 7B). When the labrum starts to develop, also this appendage expresses *Lbx/lb*. At these later stages, however, the striped segmental pattern disappears ventrally, but remains dorsally (Fig. 7C). Within the appendages, *Lbx/lb* is expressed along the ventral side of the antennae and the legs, but in the labrum this pattern is reversed onto the dorsal side, consistent with the rotation theory that suggests that the labrum rotated 180 degrees during the course of its evolution ([45] Kimm and Prpic 2006). This ventral and dorsal expression is ectodermal, and so is expression in the tips of the mandibles and the maxillae. Additional expression, however is seen in the mesoderm of the antennae and the legs (Fig. 7D-G). Expression of *Glomeris NK4/tin* first appears in the region of the invaginating stomodaeum (Fig. 7H). Later this gene is strongly expressed in the developing heart (Fig. 7I).

**Fig. 8:**
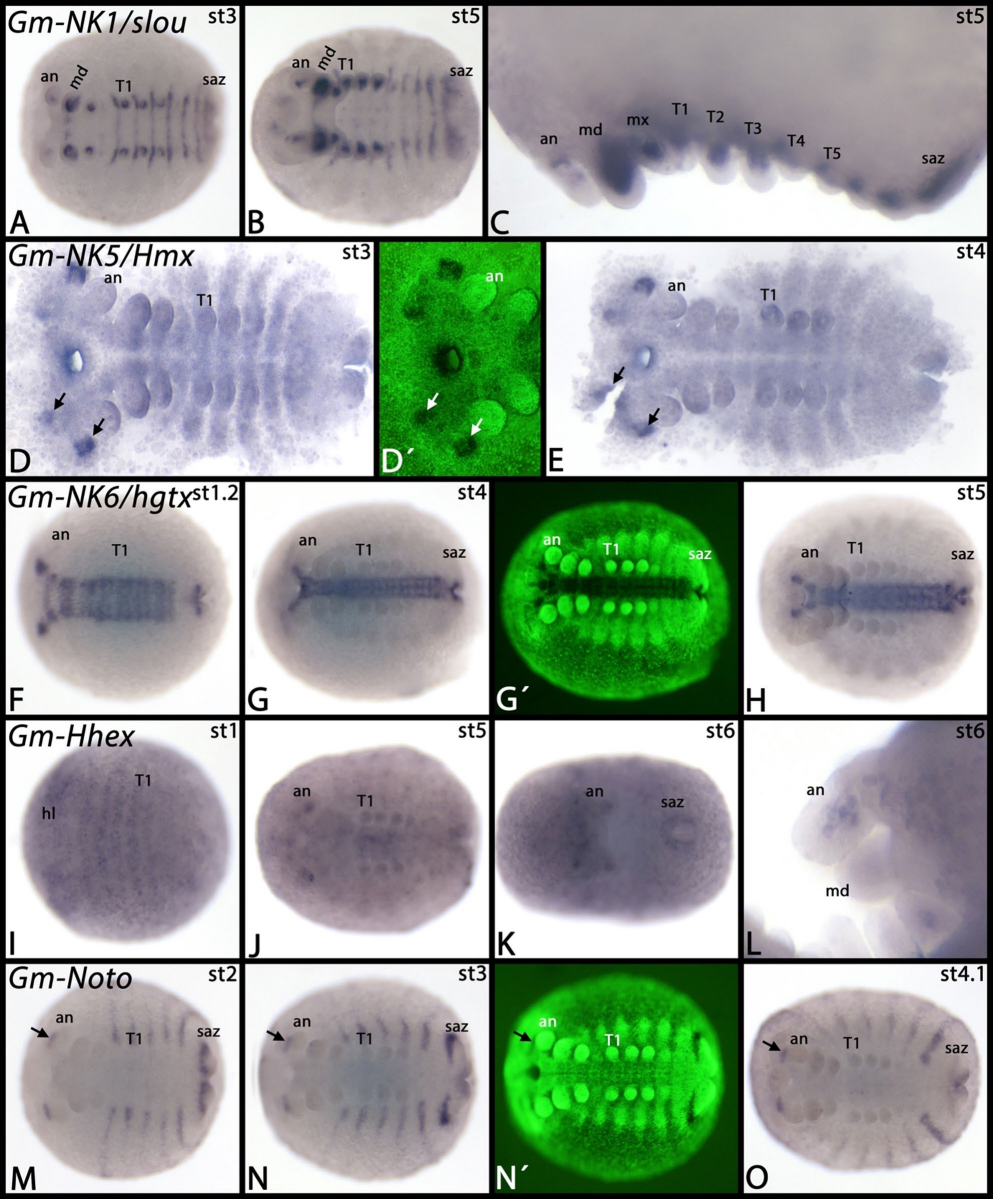
Expression of *Glomeris NK1/slou*, *NK5/Hmx*, *NK6/hgtx*, *Hhex*, and *Noto*. In all panels, anterior is to the left, except panel **L** in which anterior is up. Panel **C** represents a magnification. Arrows in panels **D** and **E** mark broad domains of expression in the head lobes. The arrows in panels **M**-**O** mark expression anterior to the base of the antennae. Panels marked with an apostrophe represent SYBR-green staining of embryos in corresponding panels. Developmental stages are indicated in the top right corner of each panel. Abbreviations: an, antenna; hl, head lobe; md, mandible; mx, maxilla; T, trunk segment; saz, segment addition zone

**Fig. 9:**
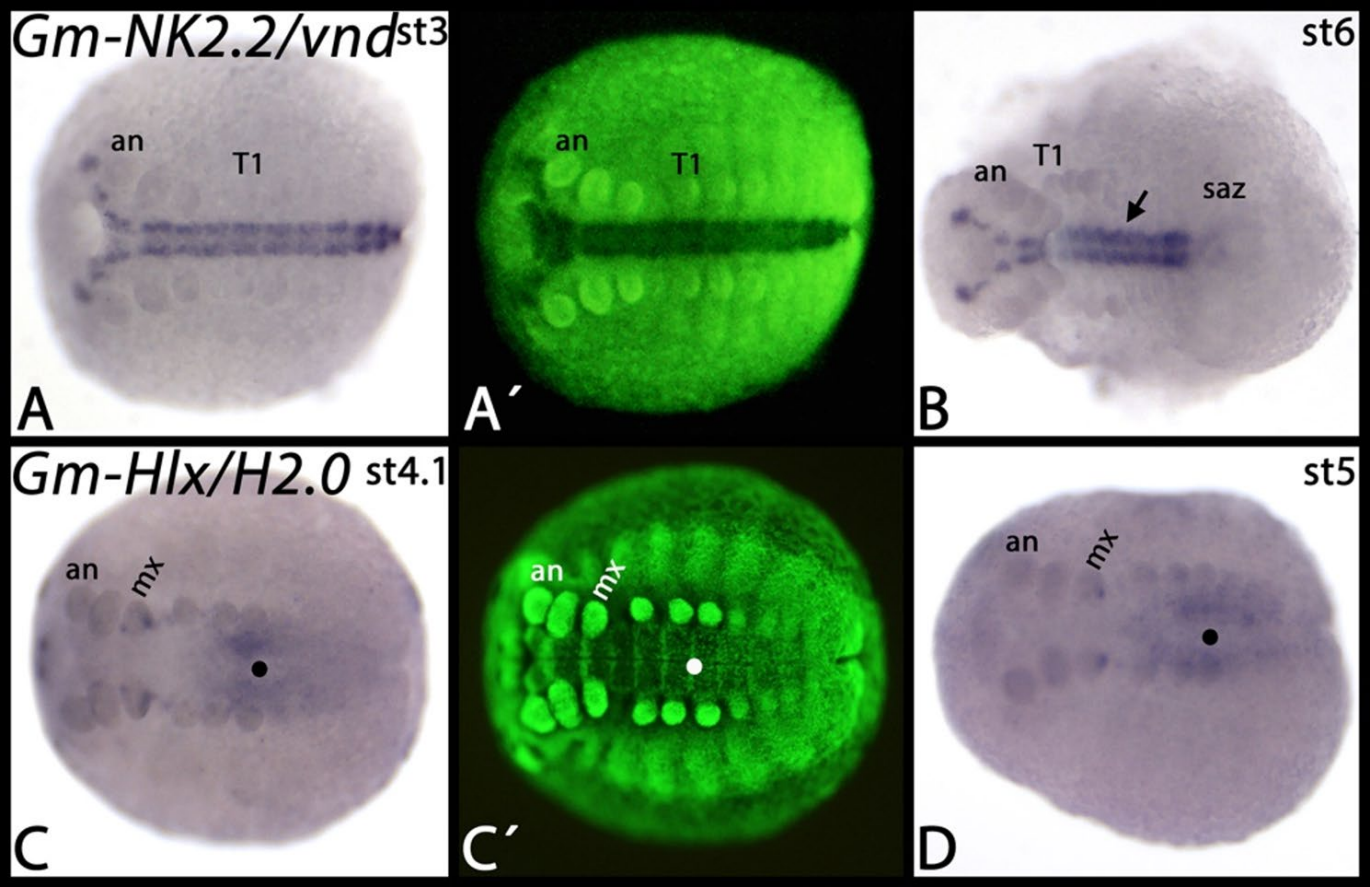
Expression of *Glomeris NK2.2/vnd* and *Hlx/H2.0*. In all panels, anterior is to the left. The arrow in panel **B** points to expression at the ventral midline. Filled circles in panels **C** and **D** mark diffuse expression surrounding the midgut. Panels marked with an apostrophe represent SYBR-green staining of embryos in corresponding panels. Developmental stages are indicated in the top right corner of each panel. Abbreviations: an, antenna; mx, maxilla; T, trunk segment; saz, segment addition zone

*Glomeris NK7* is expressed ubiquitously, but there is stronger expression in the ventral midline (Fig. 7J-L). Interestingly, the dorsal expression of *NK7* that corresponds to the maxillary segment extends into the extraembryonic tissue (Fig. 7K, L). *Glomeris NK3/bap* is expressed as distinct dots in the head lobes and in the visceral mesoderm of the trunk (Fig. 7M-O). Note the border of this expression at the interface between the postmaxillary segment and the first trunk segment (cf. expression of the visceral mesoderm marker *Glomeris FoxF* ([46] Janssen et al. 2022)). At later developmental stages, similar expression also appears in the head (Fig. 7N, O). *Glomeris NK1/slou* is expressed inside the developing appendages and as transverse segmental stripes anteriorly abutting the anterior margin of the trunk appendages (Fig. 8A-C). In the antennae, expression is weaker than in the other appendages (Fig. 8C). All expression of *NK1/slou* is likely mesodermal. *Glomeris NK5/Hmx* is expressed ubiquitously (or the probe causes background signal). Stronger expression is in the developing head lobes as four distinct domains (Fig. 8D, E). *Glomeris NK6/hgtx* is exclusively expressed in the central nervous system and in a small portion of the segment addition zone (Fig. 8F-H). *Glomeris Hhex* is first expressed ubiquitously (Fig. 8I). At later stages, specific expression is inside of some of the appendages (Fig. 8J-L), faintly in the region where the midgut will form (Fig. 8J), and as a horseshoe-shaped domain around the proctodaeum (Fig. 8K). *Glomeris Noto* is dynamically expressed in the segment addition zone (Fig. 8M, N). Expression, however, never appears in the ventral portion of the segments but instead is restricted to the posterior region of dorsal segmental units (Fig. 8M-O) (cf. [27]. Janssen et al. 2004). There are two distinct dots in the head lobes anteriorly abutting the antennae (Fig. 8M-O). *Glomeris NK2.2/vnd* is exclusively expressed in the central nervous system along the ventral midline (Fig. 9A, B). *Glomeris Hlx/H2.0* is expressed as four distinct dots in the developing maxillae, two dots in the developing labrum, and faintly in the visceral mesoderm of the trunk (Fig. 9C) (cf. expression of *Glomeris FoxF* in the developing visceral mesoderm ([46] Janssen et al. 2022)). Expression in the labrum, and the anterior two domains in the maxillae disappear at later developmental stages, but the other expression in the maxillae and in the visceral mesoderm remains (Fig. 9D).

**Fig. 10:**
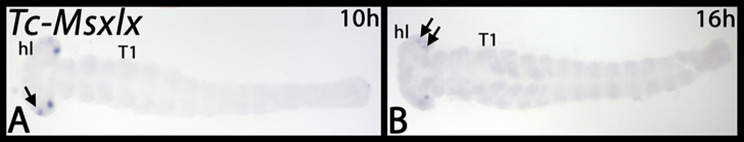
Expression of *Tribolium NK3/bap* and *Msxlx*. In all panels, anterior is to the left. Yolk has been removed, flat-mounted embryos. The arrows point to anterior expression. Developmental stages in hours after fertilization are indicated in the top right corner of each panel. Abbreviations: hl, head lobe; T, trunk segment

We also investigated the embryonic expression patterns of *Tribolium Msxlx*. This gene is exclusively expressed in few distinct dots in the head lobes (Fig. 10A, B).

## Discussion

### Lineage specific losses and duplications of NK cluster genes

All of the 16 investigated NK genes are present in a distantly related group of ecdysozoan animals, the priapulids ([47] Webster et al. 2006) (Supplementary Files 2–5). Consequently, all observed losses of these NK genes must be lineage-specific (Fig. 1A and Supplementary Files 2 and 4). The inferred losses come with the caveat that it is impossible to fully confirm the loss of a gene due to the possibilities of incomplete transcriptome and genome sequencing, as well as limitations due to the sampling of developmental stage(s) for transcriptome sequencing and the potential for sex-biased expression.

Orthologs of *Msxlx* are absent from numerous panarthropod species (Supplementary File 11). The phylogenetic distribution of this absence, however, suggests that this gene has been lost in several lineages of panarthropods independently. *Msxlx* genes have been lost in other lineages of bilaterian animals as well, such as the vertebrates, although retained in a closely related group of deuterostomes, the cephalochordates ([48] Butts et al. 2010). The repeated and independent loss of *Msxlx* in diverse lineages of bilaterian animals suggests that this gene plays “a confined role in development” that can frequently be lost during the course of evolution ([48] Butts et al. 2010). Cephalochordate *Msxlx* exhibits a transient restricted expression pattern (function) in the cerebral vesicle ([49] Lacalli and Kelly 2000, [48] Butts et al. 2010). As our in-situ hybridization results show, the expression of *Msxlx* in panarthropods that have retained this gene is also locally restricted to small regions in the developing brain (discussed below).

A striking loss is that of *Hlx*/*H2.0* in all the hitherto investigated species of modern spiders ([9] Aase-Remedios et al. 2023). We identified, however, a *Hlx*/*H2.0* ortholog in a transcriptome of the mygalomorph spider *Acanthoscurria* (Fig. 1A and Supplementary File 1), and in a transcriptome of adult tissue from the vinegaroon *Mastigoproctus* (a representative of Uropygi) ([50] Ballesteros et al. 2022) (although it was not identified in the genome ([10] Kulkarni et al. 2024)). We also identified *Hlx/H2.0* in an embryonic transcriptome of *Charinus* (a representative of Amblypygi). Likewise, we found *Hlx*/*H2.0* in a scorpion and the mite *Halotydeus* indicating that most lineages of chelicerates retained their *Hlx*/*H2.0* gene, though there may have been independent loss(es) in different lineages of chelicerates, such as in the tick *Ixodes* (Supplementary Files 6, 7 and 11).

Other inferred losses of NK genes are sparse and appear to be lineage specific, such as the absence of *Noto* and *NK7* in the tardigrade *Ramazzottius* (and possibly among tardigrades in general) (Supplementary Files 4, 5 and 11), the absence of *Hhex* in *Ixodes*, and the absence of *NK4/tin* and *NK2.1/scro* in mites (Supplementary Files 4–7 and 11) (also see [12] Klementz et al. 2025). If these suggested lineage-specific losses of NK genes are real, and do not represent sampling artifacts, then this raises three questions: why can modern spiders and ticks dispense with the function of a *Hlx/H2.0* gene, why is there no requirement of *Noto* and *NK7* in tardigrades, and why was the *Msxlx* gene lost independently in several lineages of panarthropods? In some cases, gene loss could directly be associated with the loss of a morphological structure or a developmental mechanism; for example, the loss of *NK4/tin* in mites associated with the loss of a heart in this group of chelicerates ([51] Dunlop 2019). Since data on these genes in panarthropods are relatively scarce, gene expression analysis could provide first clues about any conserved function(s) of these genes (discussed below), and why these genes were lost in some panarthropod lineages.

Another phenomenon among NK genes is gene duplication. In most panarthropods, however, gene duplication is limited and restricted to few genes. The NK genes that are most regularly duplicated are *Emx/ems* and *Msx/Dr* (Supplementary File 11) ([52] Ontano et al. 2021, [12] Klementz et al. 2025). *Emx/ems* is also duplicated in the priapulid which could suggest an ancient duplication of this gene and subsequent loss in other lineages of ecdysozoans including many panarthropods. Alternatively, and more plausibly, these may be independent duplications (and subsequent retention) of this gene, especially because several NK genes are indeed duplicated in the priapulid which would rather suggest a major duplication event in this group. The available chelicerate data, however, also suggest a duplication in their stem (Supplementary File 11) ([52] Ontano et al. 2021, [12] Klementz et al. 2025).

Like *Emx/ems*, the *Msx/Dr* gene also appears to have been duplicated in the lineage leading to chelicerates (Supplementary File 11) ([12] Klementz et al. 2025), although it is also present as two copies in the priapulid (possibly for the same reason mentioned above) and insects like *Tribolium* (but this appears to be a lineage-specific duplication within Mandibulata) ([19] Mulhair et al. 2023)). In the cases of some species of ticks and mites, one would in this scenario assume loss of one copy of *Emx/ems* (note the presence of two copies of *Emx/ems* in *Sarcoptes scabiei* ([12] Klementz et al. 2025)) and independent duplication of *Msx/Dr* in the sea spider *Pycnogonum* and in the solifuges *Gluvia dorsalis* and *Titanopuga salinarum* (Supplementary File 11) ([53] Gainett et al. 2024b, [12] Klementz et al. 2025, [54] Papadopoulos et al. 2025).

Most intriguing is the high number of duplicated NK genes in arachnopulmonate chelicerates that likely results from a whole genome duplication (WGD) in this group ([23] Schwager et al. 2017, [12] Klementz et al. 2025), but the biological reasons for the retention of these genes are unclear (discussed in [55] Sharma 2023 and [56] Munegowda et al. 2025). In this context, it has been suggested that genes that comprise higher complexity (higher number of active protein domains and cis-regulatory elements) may be retained after gene duplication more preferentially over other genes that are less complex (e.g. [57] He and Zhang 2005, [58] Guo 2017). If this is the case for panarthropod *Emx/ems* and/or *Msx/Dr* orthologs, however, has not been investigated yet.

### Conserved and diverged embryonic expression patterns of panarthropod NK genes

**Fig. 11:**
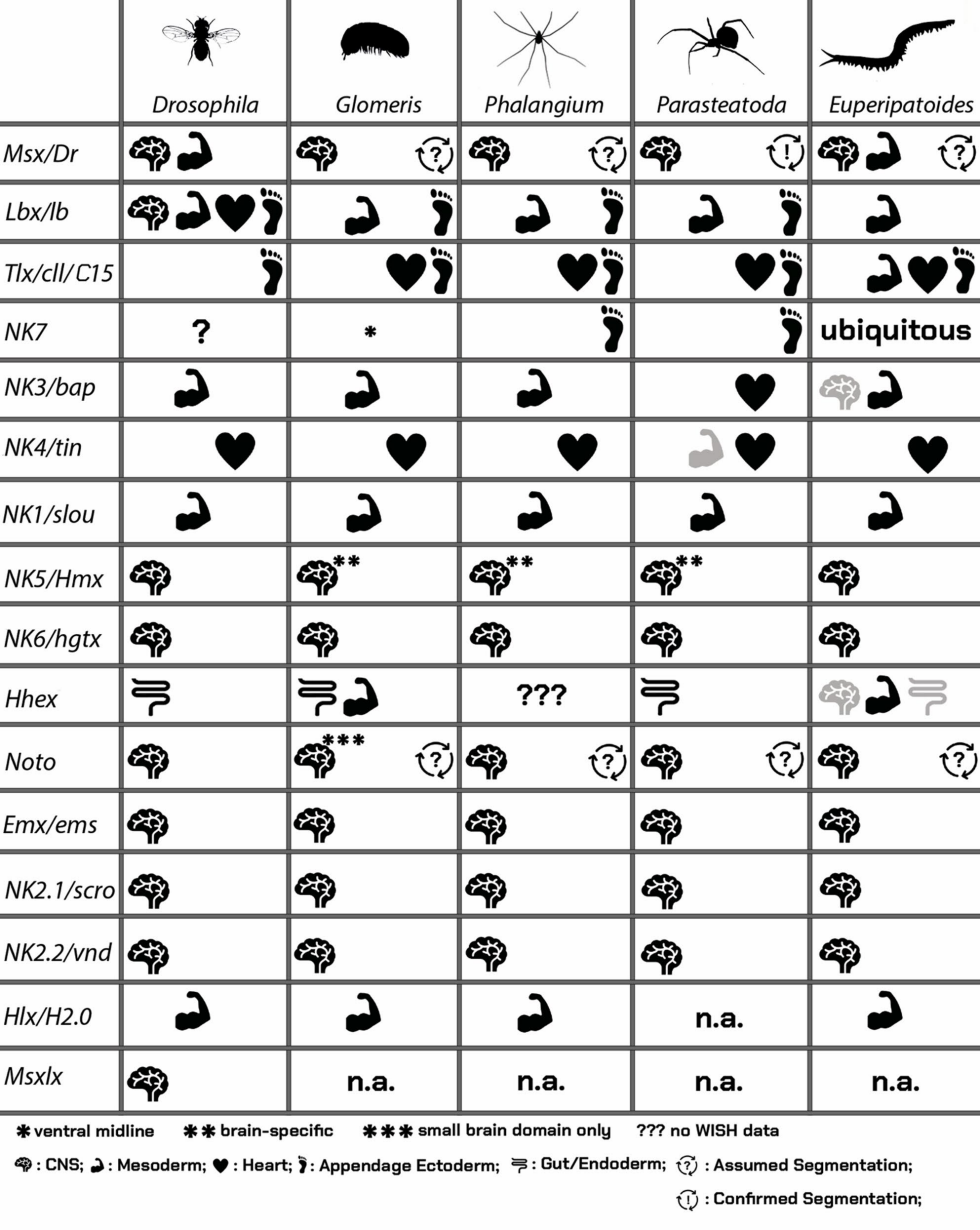
Summary of conserved and diverged expression patterns in panarthropods. Black symbols represent stronger evidence than grey symbols which represent weaker evidence. See figure for further information. Abbreviations: n.a., not applicable

*Msx/Dr* is a conserved factor of nervous system development (reviewed in [59] Ramos and Robert 2005). In all previously investigated panarthropod species, including the species addressed in this study, *Msx/Dr* is indeed expressed in the developing central nervous system (Fig. 11) ([60] Skeath 1999, [61] Wheeler et al. 2005, [62] Döffinger and Stollewerk 2010, [37] Leite et al. 2018, [7] Treffkorn et al. 2018). Here it appears to interact with another NK gene, *NK2.2/vnd* during dorsoventral patterning of the central nervous system ([63] Seibert and Urbach 2010, [62] Döffinger and Stollewerk 2010). At least in *Drosophila*, *Msx/Dr* is also transiently expressed in the developing mesoderm ([64] Bodmer et al. 1990). In the onychophoran *Euperipatoides rowelli* expression is in the limb mesoderm, but also in the ectoderm of the SAZ and later as broad transverse stripes in the formed segments ([7] Treffkorn et al. 2018). In this context it is interesting to note that at least one of the two paralogs of *Msx/Dr* in the spider *Parasteatoda* (*Msx-1*) has a role in segmentation and is expressed in the characteristic pattern of transverse stripes in the SAZ, newly formed segments, and rings in the germ disc (Akiyama-Oda and Oda 2020) [43]. Likewise, in the myriapod *Glomeris* and the harvestman *Phalangium*, *Msx/Dr* is expressed as stripes in the SAZ and newly formed segments, and later also in the developing nervous system ([65] Dove 2003, [37] Leite et al. 2018). Functions in central nervous system development and segmentation thus appear to be the conserved features of *Msx/Dr* in panarthropods (Fig. 11).

*Lbx/lb* is expressed in the mesoderm and the ventral sector of the developing appendages of all hitherto investigated arthropods ([66] Maqbool et al. 2006, [67] Cande et al. 2009, [9] Aase-Remedios et al. 2023) (Fig. 11). In the onychophoran *Euperipatoides rowelli*, *Lbx/lb* also is expressed in the developing head ([7] Treffkorn et al. 2018). In *Drosophila*, *Lbx/lb* genes are known to play an additional role in nervous system differentiation and heart development ([68] Jagla et al. 1994, [69], Jagla et al. 1997, [70] Jagla et al. 1998). Interestingly, however, the heart expression is not conserved in other arthropods (discussed below). Expression in the mesoderm and the ventral sector of the developing limb ectoderm thus appear to represent the conserved ancestral expression features of this gene (Fig. 11).

Expression of *Tlx/cll/C15* in the tips of the developing limbs is conserved among arthropods ([71] Janssen 2017a, [9] Aase-Remedios et al. 2023), and may be under control of Egfr-signaling, as described for *Drosophila* (discussed in [71] Janssen 2017a). In *Euperipatoides rowelli*, *Tlx/cll/C15* is expressed in subsets of the developing limb and trunk mesoderm, and at later developmental stages, also in the tips of the developing limbs ([7] Treffkorn et al. 2018); note that in *Euperipatoides kanangrensis* expression of *Tlx/cll/C15* in the heart has been reported ([15] Janssen et al. 2014). In previously investigated arthropods except *Drosophila*, *Tlx/cll/C15* is also dominantly expressed in the developing heart ([30] Grossmann and Prpic 2012, [71] Janssen 2017a, [9] Aase-Remedios et al. 2023). Thus, it is likely that expression in the heart and the tips of the appendages are ancestral and conserved features and that the lack of expression in the heart in *Drosophila* represents a derived state (Fig. 11).

The ancestral function (if any) of *NK7* in panarthropods is unclear (Fig. 11). Expression of *NK7* in *Drosophila* has superficially been investigated using Northern analysis on a pooled RNA extraction of mixed stages of embryos ([72] Sakoyama et al. 2002) and enhancer trap lines ([73] Kockel et al. 2019). In addition, several gene expression analysis attempts are reported by the Berkeley *Drosophila* Genome Project ([74] Tomancak et al. 2002, [75] Tomancak et al. 2007, [76] Hammonds et al. 2013); (https://insitu.fruitfly.org/cgi-bin/ex/report.pl?ftype=10%26ftext=FBgn0024321 (as per 14th of October 2024)), but with somewhat inconclusive and contradictory outcomes. To the best of our knowledge, there are no published data on *NK7* expression in any other insect or crustacean. In the chelicerates *Parasteatoda* and *Phalangium*, *NK7* genes are expressed in a subset of cells in the limb ectoderm and the developing head lobes ([9] Aase-Remedios et al. 2023). This pattern, however, is not conserved in the myriapod *Glomeris* or the onychophoran *Euperipatoides kanangrensis*.

The expression of panarthropod *NK3/bap* is conserved in the visceral trunk mesoderm ([77] Azpiazu and Frasch 1993, [78] Azpiazu et al. 1996, [7] Treffkorn et al. 2018, [9] Aase-Remedios et al. 2023) (Fig. 11). One exception is the spider in which *NK3/bap* is expressed in the developing heart (not seen in any other species) ([9] Aase-Remedios et al. 2023).

*NK4/tin* is a conserved factor of panarthropod heart development, and to the best of our knowledge we are not aware of any previously investigated species in which *NK4/tin* is not prominently expressed in the developing heart ([77] Azpiazu and Frasch 1993, [42] Janssen and Damen 2008, [7] Treffkorn et al. 2018, [9] Aase-Remedios et al. 2023). Interestingly, the gene is also expressed at the anterior edge of the head lobes in *Parasteatoda* and another previously investigated spider, *Cupiennius salei*, and this *NK4/tin* expressing tissue later fuses with the opisthosomal part of the heart tissue to form one continuous tube ([42] Janssen and Damen 2008, [9] Aase-Remedios et al. 2023). *NK4/tin* is thus a highly-conserved factor of panarthropod heart development (Fig. 11).

*NK1/slou* likely represents a conserved mesodermal gene in panarthropods (Fig. 11). In line with this hypothesis, in both the onychophoran *Euperipatoides rowelli* ([7] Treffkorn et al. 2018) and the arthropods investigated in this study, *NK1/slou* is expressed in the developing mesoderm. In *Drosophila*, *NK1/slou* is involved in the development of a subset of somatic muscle founder cells ([79] Knirr et al. 1999).

The predominant expression (and likely function) of *NK5/Hmx* in the developing head appears to be the conserved feature of this gene in Panarthropoda (Fig. 11). In the onychophoran *Euperipatoides rowelli*, *NK5/Hmx* is first exclusively expressed in the developing head lobes, and later also in the mesoderm of some (but not all legs) and the distal mesoderm of the frontal appendages (the antennae of the onychophoran), and faintly in the developing nervous system ([7] Treffkorn et al. 2018). In *Drosophila*, and the arthropods investigated in this study, *NK5/Hmx* is predominantly expressed in the developing brain, and knock-down in *Drosophila* leads to head defects including the ablation of the eyes ([80] Quiquand et al. 2021). In *Drosophila*, *Nk5/Hmx* is also expressed in the developing ventral nerve cord ([81] Wang et al. 2000).

In all hitherto investigated panarthropod species, *NK6/hgtx* is expressed in the developing central nervous system showing that the function of this NK gene is highly conserved in this group of animals (Fig. 11), and indeed this functional conservation may even date back to the ancestor of Bilateria ([82] Cheesman et al. 2004).

Data on *Hhex* are relatively sparse, but suggest a possibly conserved function in the development of the endodermal midgut. In the onychophoran *Euperipatoides rowelli*, however, *Hhex* is expressed in the mesoderm of the appendages, and at later developmental stages also in the central nervous system ([20] Treffkorn and Mayer 2019). In the spider *Parasteatoda*, the detected expression may be associated with the developing endoderm, but there is no mesodermal expression, and in the myriapod *Glomeris*, expression is restricted to the late mesoderm of the appendages (as in the onychophoran), the midgut, and a horseshoe-shaped domain around the proctodaeum. Interestingly, in *Drosophila*, *Hhex* is exclusively expressed in the midgut at relatively late developmental stages (Berkeley *Drosophila* Genome Project ([74] Tomancak et al. 2002, [75] Tomancak et al. 2007, [76] Hammonds et al. 2013); https://insitu.fruitfly.org/cgi-bin/ex/report.pl?ftype=1%26ftext=FBgn0038852; (as per 23rd of September 2024), and loss of *Hhex* activity leads to a disturbed metabolism due to its effects in the fat body ([83] Pendse et al. 2013). In this respect, additional evidence comes from a study on the mitten crab *Eriocheir senensis* in which *Hhex* is upregulated upon starvation indicating its function in the hepatopancreas ([84] Liu et al. 2023). It is thus likely that the midgut expression/function of *Hhex* is at least conserved in arthropods, but possibly not onychophorans (Fig. 11). In vertebrates, *Hhex* is a conserved endodermal determinant (reviewed in [85] Jackson et al. 2023), suggesting that the lack of *Hhex* expression in definite endodermal structures and the described expression patterns in onychophorans would represent derived patterns. However, late endodermal tissues have not been described in great detail yet in any onychophoran species, although the expression of early endoderm markers have been described ([86] Janssen and Budd 2017, [34] Janssen and Budd 2024). It is thus possible that some of the described expression of *Hhex* in the onychophoran *Euperipatoides rowelli* may be correlated with the development of the endoderm.

Expression of *Noto* in the developing ventral nervous system appears to be conserved, but the dynamic expression of *Noto* in the SAZ of the here investigated arthropods resembles that of the canonical pair-rule genes (PRGs) and other segmentation genes such as *Notch* (*N*) and *Delta* (*Dl*) (e.g. [87] Stollewerk et al. 2003, [88] Damen et al. 2005, [89] Choe et al. 2006, [90] Chipman and Akam 2008, [91] Janssen et al. 2011, [92] Eriksson et al. 2013). In onychophorans, however, expression of the PRGs, *N*, and *Dl* is restricted (if at all expressed in the SAZ) to a small domain ([22] Janssen and Budd 2013, [93] Janssen and Budd 2016), and so is *Noto* (Fig. 2). This implies that *Noto* acts on the level of the PRGs and N/Dl-signaling during posterior segment addition and/or posterior elongation, but testing this hypothesis would require functional studies. The lack of segmentation-related expression of *Noto* in *Drosophila*, however, could be associated with its derived mode of development, as it is also the case for *N* and *Dl*. In any case, *Drosophila Noto* is expressed in the ventral nervous system and the central brain glia (Berkeley *Drosophila* Genome Project ([74] Tomancak et al. 2002, [75] Tomancak et al. 2007, [76] Hammonds et al. 2013)); https://insitu.fruitfly.org/cgi-bin/ex/report.pl?ftype=1%26ftext=FBgn0038592#RE58281 (as per 23rd of September 2024). Expression in the ventral nervous system and the role in segmentation could thus represent the ancestral expression/function(s) of this gene in panarthropods (Fig. 11).

*Emx/ems* is a highly conserved factor of nervous system development among the Bilateria and possibly even Metazoa as a whole (discussed in [20] Treffkorn and Mayer 2019). In the onychophoran *Euperipatoides rowelli* ([20] Treffkorn and Mayer 2019) – but not in *Euperipatoides kanangrensis* ([94] Janssen 2017b) – expression has also been reported in a subset of the limb mesoderm. Since there are no reports about mesodermal expression of *Emx/ems* in any of the previously studied arthropods ([95] Walldorf and Gehring 1992, [96] Simonnet et al. 2006, [97] Birkan et al. 2011, [37] Leite et al. 2018, [98] Nazar et al. 2022, [9] Aase-Remedios et al. 2023), it is either that the situation in *Euperipatoides rowelli* is derived, or that it may represent a staining artefact. Further investigations should be conducted in the future to address this question. Interestingly, the two NK genes *NK6/hgtx* and *Emx/ems* both are involved in dorsoventral patterning of the brain in *Drosophila*, a function that may be conserved in panarthropods as well ([99] Seibert et al. 2009) (Fig. 11).

*NK2.1/scro* is a conserved factor of bilaterian head nervous system development ([100] Takacs et al. 2002, [101] Tessmar-Raible et al. 2007, [102] Bishop et al. 2013, [103] Martín-Durán and Hejnol 2015, [104] Yoo et al. 2020). Among the investigated panarthropods, at least in *Drosophila* and *Euperipatoides rowelli*, (but not *Euperipatoides kanangrensis* ([105] Janssen 2017c)) *NK2.1/scro* is also expressed in the ventral nerve cord ([106] Zaffran et al. 2000, [20] Treffkorn and Mayer 2019). In *Parasteatoda*, *Phalangium*, *Glomeris*, the centipede *Strigamia*, and the beetle *Tribolium*, however, there is no such expression ([107] Hunnekuhl and Akam 2014, [105] Janssen 2017c, [108] Posnien et al. 2011, [109] Medina-Jiménez et al. 2024b). We conclude that the conserved expression/function of *NK2.1/scro* lies in the development of the panarthropod head nervous system (Fig. 11).

*NK2.2/vnd* is a conserved factor of ventral nervous system development in panarthropods ([110] Skeath et al. 1994, [61] Wheeler et al. 2005, [37] Leite et al. 2018, [7] Treffkorn et al. 2018, [111] Treffkorn et al. 2022). In *Drosophila*, *NK2.2/vnd*, *Msx/Dr*, *Gsx/ind* and *Egfr* are called columnar genes as they pattern the dorsoventral axis of the central nervous system (reviewed in [60] Skeath 1999), and this function appears to be conserved in the beetle *Tribolium* ([61] Wheeler et al. 2005, [112] Biffar and Stollewerk 2015), and may be ancestral to bilaterians (reviewed in [113] Cornell and Ohlen 2000) (Fig. 11).

The data on *Hlx/H2.0* presented in this paper are consistent with a function in mesoderm and visceral musculature development. Unfortunately, we could not detect any specific expression of *Hlx/H2.0* in the tarantula *Acanthoscurria* which would have been interesting given that *Hlx/H2.0* was lost in true spiders. In *Drosophila*, *Hlx/H2.0* is expressed in the visceral musculature including a sheet of visceral muscles surrounding the midgut and as segmental patches in the ventral epidermis ([114] Barad et al. 1988) (Berkeley *Drosophila* Genome Project ([74] Tomancak et al. 2002, [75] Tomancak et al. 2007, [76] Hammonds et al. 2013)); https://insitu.fruitfly.org/cgi-bin/ex/report.pl?ftype=1%26ftext=FBgn0001170 (as per 24th of September 2024). From the available data, we conclude that *Hlx/H2.0* is a conserved marker of the developing panarthropod visceral mesoderm (Fig. 11).

Though there is limited available information on *Msxlx* expression, its ancestral role may be correlated with a function in brain development. In *Drosophila*, *Msxlx* is expressed in the procephalic ectoderm (likely some isolated neuroblasts within this region), and in the ventral nerve cord (Berkeley *Drosophila* Genome Project ([74] Tomancak et al. 2002, [75] Tomancak et al. 2007, [76] Hammonds et al. 2013)); https://insitu.fruitfly.org/cgi-bin/ex/report.pl?ftype=1%26ftext=FBgn0038833 (as per 19th of September 2024)), and at least the expression in the developing brain appears to be conserved in the beetle *Tribolium* (Fig. 10A, B). Based on this new information it is likely that the expression of *Msxlx* in the brain is conserved, at least in higher insects (Fig. 11). Whether this is the ancestral function of *Msxlx* in arthropods, however, remains unclear due to the lack of comparative data. Expression of *Msxlx* in the ventral nerve cord as reported for *Drosophila* (but only in one of two independent experiments) (see Berkeley *Drosophila* Genome Project data) was not seen in *Tribolium* (Fig. 10A, B). It could thus be that the nerve cord data reported for *Drosophila* represent a possible artefact/contamination.

While it appears clear for some of the here investigated NK genes what their conserved patterns/functions in panarthropods are, this remains currently unclear for others. We therefore suggest additional investigation into the expression and function of these genes in species in which they have been retained.

### NK-gene clustering and gene order in the last common ancestor of bilateria

In arthropods, 16 NK genes have been identified as part of clusters, nine core genes of the NK cluster (NK) (that were also present in the last common ancestor of bilaterians (e.g. [115]. Garcia-Fernàndez 2005, [116] Wotton et al. 2009, [117] Hui et al. 2012, [1] Ferrier 2016, [6] Chan et al. 2015)), three additional linked genes (NKL), and four genes that form a separate cluster (NK2) (Fig. 1B) ([9] Aase-Remedios et al. 2023, [10] Kulkarni et al. 2024, [11] Aase-Remedios et al. 2025, [12] Klementz et al. 2025).

**Fig. 12:**
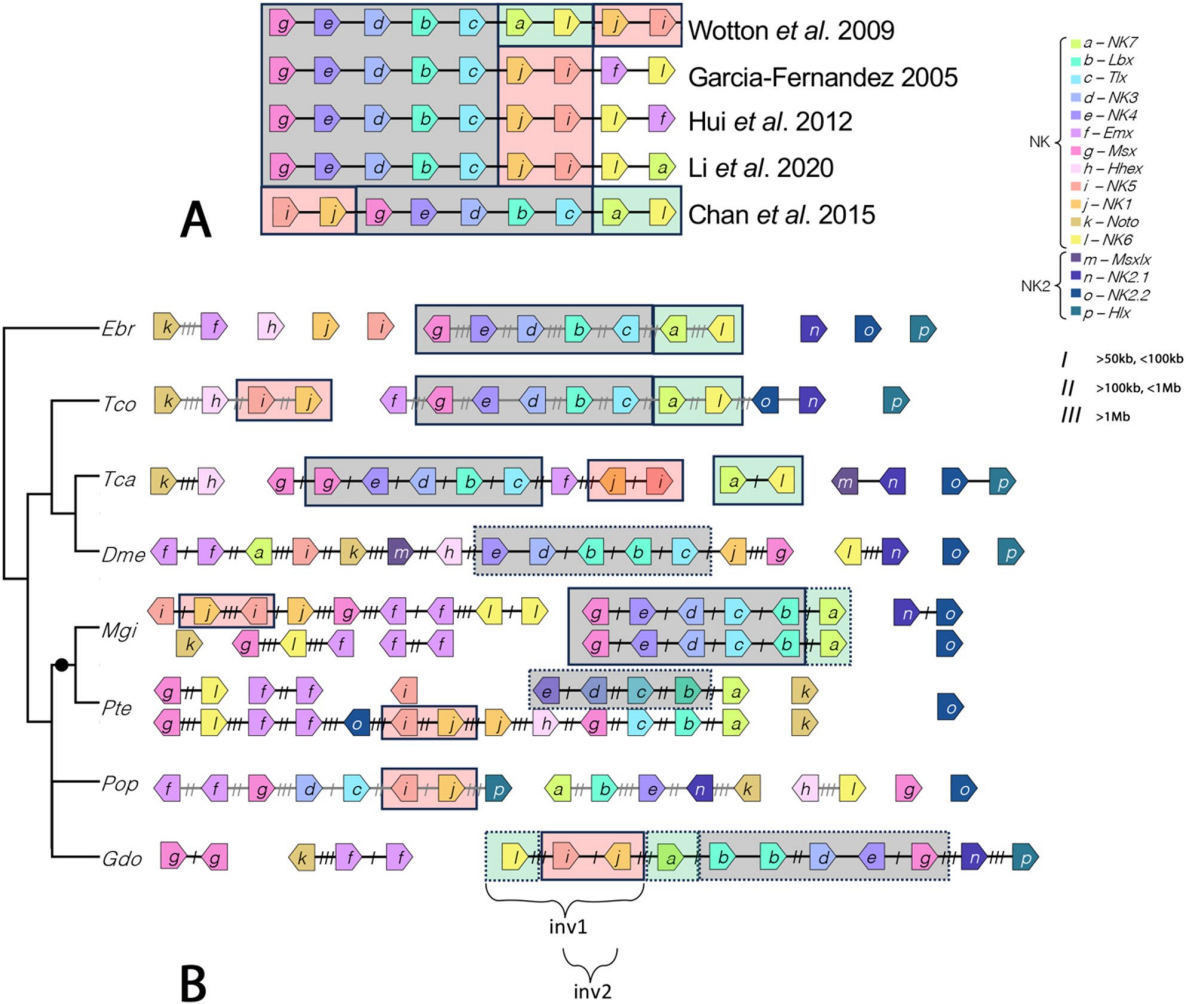
NK-gene clustering. **A** Predicted NK core cluster in the last common ancestor of Bilateria. Note the agreement that five of the genes are in conserved order (underlain in grey). Also note agreement on the conserved *NK1/slou* + *NK5/Hmx* tandem (underlain in red) [116] Wotton et al. (2009) and [6] Chan et al. (2015) also agree on the *NK7* + *NK6/hgtx* tandem (underlain in green). **B** Clustering of core NK genes in some relevant example panarthropod species. The two predicted inversion events in *Gluvia* that would restore the synteny suggested by [116] Wotton et al. (2009) are indicated. The full circle marks arachnopulmonate species with a whole genome duplication. Number of slashes between the NK genes indicate distance on the chromosome (also see in-figure legend). Species abbreviations: Dme, *Drosophila melanogaster*; Ebr, *Epiperipatus broadwayi*; Gdo, *Gluvia dorsalis*; Mgi, *Mastigoproctus giganteus*; Pop, *Phalangium opilio*; Pte, *Parasteatoda tepidariorum*; Tca, *Tribolium castaneum*; Tco, *Trigoniulus corallinus*

Despite the agreed number of nine core genes in the NK cluster, the predicted order of these genes in the last common ancestor of Bilateria is contentious (and partially even different genes were incorporated into this core) (e.g. [115] Garcia-Fernàndez 2005, [116] Wotton et al. 2009, [117] Hui et al. 2012, [6] Chan et al. 2015, [8] Li et al. 2020) (summarized in Fig. 12A). The presence and order of the five NK genes *Msx/Dr-NK4/tin-NK3/bap-Lbx/lb-Tlx/C15/cll* is common (Fig. 12A, underlined in grey) [116]. Wotton et al. (2009) and [6] Chan et al. (2015) suggest that these five genes are followed by *NK7* and *NK6/hgtx* (Fig. 12A, underlined in green). In the view of [116] Wotton et al. (2009) these are followed by *NK1/slou* and *NK5/hmx* (Fig. 12A, underlined in red), while in the view of [6] Chan et al. (2015) the latter two are placed in reversed order in front of *Msx/Dr* (Fig. 12A) [115] Garcia-Fernàndez (2005) and [117] Hui et al. (2012) suggest that *NK6/hgtx* and *Emx/ems* follow the five NK core genes albeit in reverse order relative to one another. In this scenario, however, Garcia-Fernàndez (2005) [115] does not define *Emx/ems* and *NK6/hgtx* as part of the core at all (unlike depicted in Fig. 12A). Some authors indeed consider *Emx/ems* as part of the core cluster, while others consider *NK7* instead (Fig. 12A).

The reason for these uncertainties is the relatively low number of species that have been investigated with respect to NK-gene clustering thus far, either because informative genomic data were unavailable in the past, or because this question has not been addressed. The recent availability of high-quality (near) chromosome-level genomic data sets, and the increased interest in NK genes and their clustering, however, may help answering this open question. Among arthropods (and their relatives within Ecdysozoa), we now possess a broader understanding of the genome structure of species representing multiple orders of chelicerates ([53] Gainett et al. 2024b, [10] Kulkarni et al. 2024, [11] Aase-Remedios et al. 2025, [12] Klementz et al. 2025, [54] Papadopoulos et al. 2025), especially spiders (summarized in [9] Aase-Remedios et al. 2023), myriapods ([118] Chipman et al. 2014, [119] Qu et al. 2020), crustaceans ([120] Colbourne et al. 2011, [121] Bett et al. 2024), insects (reviewed in [122] Hotaling et al. 2021), and onychophorans ([123] Sato et al. 2023) (Fig. 12B).

These data confirm the predicted core of five conserved genes (underlined in grey in Fig. 12A) and their conserved order within the NK cluster as we find this order in the insect *Tribolium*, the myriapod *Trigoniulus corallinus*, and the onychophoran *Epiperipatus broadwayi* (Fig. 12B). In the stem of chelicerates, an inversion of *Lbx/lb* and *Tlx/C15/cll* appeared (seen in many chelicerates) (e.g. [9]. Aase-Remedios et al. 2023, [12] Klementz et al. 2025). Beyond that, however, the content and order of the five genes is also conserved in the chelicerate *Mastigoproctus* (Fig. 12B). Importantly, this is the case for both copies of the cluster (after the WGD in Arachnopulmonata) making it very likely that this order was also conserved in the last common ancestor of Arachnopulmonata ([10] Kulkarni et al. 2024). Slightly derived patterns of this five gene core are present in multiple spiders, the mite *Sarcoptes* and the solifuge *Gluvia* (Fig. 12B) ([12] Klementz et al. 2025). The only differences are the apparent loss of *NK4/tin* in *Sarcoptes* and *Tlx/C15/cll* in *Gluvia*, alongside a duplication of *Lbx/lb* in the latter ([12] Klementz et al. 2025). Either *NK1/slou* or *NK7* follows *Tlx/C15/cll* (or *Lbx/lb* in chelicerates due to lineage-specific inversion). Only the arrangement in *Drosophila* supports *NK1/slou* following *Tlx/C15/cll*, but multiple cases show *NK7* following *Tlx/C15/cll* (*Mastigoproctus*, *Epiperipatus*, multiple spiders including *Parasteatoda*, *Phalangium*, *Trigoniulus*, *Odiellus spinosus*, and *Gluvia* (mind the inversion of *Lbx/lb* and *Tlx/C15/cll* in chelicerates)) (Fig. 12B) ([9] Aase-Remedios et al. 2023, [11] Aase-Remedios et al. 2025, [12] Klementz et al. 2025). We therefore conclude that *NK7* likely followed *Tlx/C15/cll* in the bilaterian ancestor. Multiple species have NK gene arrangements where *NK6/hgtx* follows *NK7* (the onychophoran *Epiperipatus*, the myriapod *Trigoniulus*, the insect *Tribolium*, and the spider *Argiope bruennichi*) (Fig. 12B) ([9] Aase-Remedios et al. 2023). We find evidence for *NK1/slou* and *NK5/hmx* following *NK7* and *NK6/hgtx*, as suggested by [116] Wotton et al. (2009), in the spider *Argiope*, the tick *Sarcoptes* (but note that there is no *NK7* in this species and instead *NK1/slou* and *NK5/hmx* follow *NK6/hgtx*) and the solifuge *Gluvia* (Fig. 12B) ([9] Aase-Remedios et al. 2023, [12] Klementz et al. 2025). Interestingly, in the latter, two inversions (first of *NK6/hgtx* + *NK5/hmx* + *NK1/slou* and then of *NK5/hmx* + *NK1/slou*) would fully restore the order and content suggested by [116] Wotton et al. (2009) (Fig. 12A, B). We then investigated the alternative order suggested by [6] Chan et al. (2015), *NK1/slou* and *NK5/hmx* followed by *Msx/Dr*. Moderate evidence for this gene order comes from ticks (although the order of *NK1/slou* and *NK5/hmx* is reversed in *Sarcoptes*) ([12] Klementz et al. 2025), the spider *Latrodectus elegans* ([9] Aase-Remedios et al. 2023) and the pycnogonid *Pycnogonum* (albeit in both cases with *Hhex* between *Msx/Dr* and *Nk1/slou*+*NK5/hmx*). Generally, the multiple copies of *Msx/Dr* in the investigated species complicate the analysis because random placement of one copy of *Msx/Dr* next to *NK1/slou* and/or *NK5/hmx* becomes statistically more likely than for NK genes that are not present as multiple copies. Finally, we did not find any compelling evidence for *Emx/ems* and/or *NK6/hgtx* following *NK5/hmx*, as suggested by [115] Garcia-Fernandez (2005) and [117] Hui et al. (2012).

Based on our analysis, we therefore conclude that the clustering and gene order suggested by [116] Wotton et al. (2009) is the most parsimonious for the bilaterian ancestor. As mentioned above, the most intact NK cluster is thus found in the solifuge *Gluvia* in which two inversion events would restore the gene order to the predicted order in the bilaterian ancestor (indicated in Fig. 12B). Similarly, we find the order of seven of the nine genes intact in the onychophoran *Epiperipatus*. In this species only *NK1/slou* and *NK5/hmx* are possibly not clustered, but its genome is not fully assembled and annotated (both *NK1/slou* and *NK5/hmx* are on small separate scaffolds) (Fig. 12B). Thus, the two un-clustered genes in question could indeed follow the seven clustered genes in the predicted order.

### NK genes and CRE-shuffling

In the context of this paper, we aim to better understand why the clustering of some genes is highly conserved since the last common ancestor of Bilateria, while others dispersed generally, or in different lineages of bilaterian animals including arthropods. The widely accepted and most parsimonious explanation for conserved gene clustering is that these genes share common cis-regulatory elements (CREs). Such CREs can be global cluster elements that exert control over all (or many) genes of a given cluster constraining its overall integrity over the course of evolution. CREs can also be short-range elements that exert control over one or a limited number of genes in tandem within a given cluster constraining the gene order within a cluster at a smaller scale (reviewed in [124] Pearson et al. 2005, [125] Srinivasan and Mishra 2020, [126] Panigrahi and O’Malley 2021).

*NK4/tin* and *Lbx/lb* are classically (from our knowledge of *Drosophila* development) associated with expression and function in the developing dorsal tube, the heart of panarthropods. In the beetle *Tribolium*, however, *Lbx/lb* is not expressed in the heart, and instead its neighboring gene in the cluster, *Tlx/cll/C15*, is ([67] Cande et al. 2009). This is due to an inversion that places the heart-specific enhancer controlling *Lbx/lb* expression in *Drosophila* in a position controlling *Tlx/cll/C15* in *Tribolium*. Due to the highly-related nature of the NK genes, one (*Tlx/cll/C15*) seems to be able to assume the function of the other (*Lbx/lb*), which may provide the flexibility to allow for enhancer shuffling ([67] Cande et al. 2009).

From our comparison of NK gene expression, the arrangement in *Tribolium* likely represents the ancestral state, as there is expression of *Tlx/cll/C15* in the heart of all other investigated panarthropod species (*Glomeris*, *Parasteatoda*, *Phalangium*, and *Euperipatoides*), and at the same time there is no such heart expression of *Lbx/lb* genes in these species ([127] Oliveira et al. 2014, [71] Janssen 2017a, [9] Aase-Remedios et al. 2023) (Fig. 7A-C). Our work thus highlights another of many examples in which the development of *Drosophila* represents the exception from the rule rather than a representative feature of arthropods. It also highlights the crucial need to consider all paralogs of a given ortholog and also non-orthologous closely-related genes in comparative gene expression (and function) studies, especially when investigating linked/clustered genes. The example presented by [67] Cande et al. (2009) likely does not represent an isolated case. In this context, we find evidence supporting another potential CRE-shuffling/replacement event. In the spider *Parasteatoda*, both *NK4/tin* (the conserved heart-marker) and *NK3/bap* are expressed in the developing heart ([9] Aase-Remedios et al. 2023). The latter, however, is not expressed in the heart of any of the other investigated panarthropod species (Fig. 11) ([7] Treffkorn et al. 2018, [9] Aase-Remedios et al. 2023). In analogy to the situation described above for *Lbx/lb* and *Tlx/cll/C15* in *Drosophila* and *Tribolium* ([67] Cande et al. 2009), we speculate that inversion events and thus a shift of a heart-specific enhancer within the conserved tinman gene complex (Tin-C) may underpin this difference in expression of *NK3/bap* in the spider. Without elaborate genetic analysis of the Tin-C region and subsequent transgenic experiments, however, we cannot elaborate the exact events that led to the expression of *NK3/bap* in the spider heart. Given the fact that there are likely several heart-specific enhancers in the Tin-C locus of the spider, as it is the case for insects (either ancestrally correlated with the expression of *NK4/tin* or *Tlx/cll/C15*), we also do not know which enhancer controls the expression of *NK3/bap*, further complicating the situation. One likely scenario that could have resulted in the activation of *NK3/bap* in the heart is the inversion that put *Tlx/cll/C15* next to *NK3/bap* in chelicerates including the spider (Fig. 12B) ([9] Aase-Remedios et al. 2023). In this scenario, the same heart-specific enhancer that drives *Tlx/cll/C15* could also drive expression of *NK3/bap*. Although *NK3/bap* lies next to *Tlx/cll/C15* in the harvestman *Phalangium*, *NK3/bap* is not expressed in the heart in this species ([9] Aase-Remedios et al. 2023). This could support the hypothesis that a *NK4/tin*-specific heart enhancer drives the expression of *NK3/bap* in the heart of the spider because in the harvestman the Tin-C locus is dispersed and *NK4/tin* is not clustered with *NK3/bap* and *Tlx/cll/C15* any longer (Fig. 12B). Further insight into this matter could come from gene expression analysis of Tin-C genes in the whip scorpion *Mastigoproctus* which shares (in one copy) the same gene arrangement as the spider *Parasteatoda* (Fig. 12B).

#### The ancestral function of NK genes and the Cambrian explosion

Interestingly, many of the clustered NK genes appear to play a function in mesoderm development and differentiation in bilaterian animals ([128] Balavoine 1996, [129] Dietrich et al. 1998, [130] Houzelstein et al. 1999, [131] Brohmann et al. 2000, [132] Saudemont et al. 2008) including panarthropods ([70] Jagla et al. 1998, [79] Knirr et al. 1999, [7] Treffkorn et al. 2018). This led to the idea that the radiation of NK genes may have occurred at the same time as, and even enabled, the evolution of the mesoderm and the massive radiation of animal life during the Cambrian explosion ([133] Holland 2015). This view, however, was mainly based on comparative data between vertebrates and the fly *Drosophila* ([133] Holland 2015), and addressed mostly those of the clustered NK genes that indeed are expressed in the mesoderm in these animals.

Our data (and those of others published in recent papers), however, show that many of these “mesodermal” NK genes are conserved factors of nervous system development, and others are likely conserved factors of endoderm development (summarized in Fig. 11). The interpretation of NK data by [133] Holland (2015) as fundamentally mesodermal was likely hindered by the lack of comparative expression data or genomic resources from most groups of animals at the time, especially with respect to many of the NK genes that are not predominantly expressed in the mesoderm such as *Noto* and *NK5/Hmx*. While the argument that the Cambrian radiation may be (partially) enabled by the expansion of the NK cluster was rather speculative at the time ([133] Holland 2015), the additional data now available render it unlikely. As it appears, clustered NK genes are involved in the development of all three germ layers, the ectoderm, the endoderm, and the mesoderm. Nevertheless, the expansion of gene families by gene duplication on all levels (tandem, chromosome, and whole genome) may indeed have contributed to the radiation of animals during the Cambrian explosion, but we now see no evidence for a direct and/or specific connection between the NK genes, the evolution of the mesoderm, and the Cambrian radiation based on the currently available data.

## Electronic Supplementary Material

Below is the link to the electronic supplementary material.

Supplementary Material 1: Alignment CoreTree.

Supplementary Material 2: CoreTree + *Priapulus caudatus*. Bayesian analysis using MrBayes applying three million cycles for the Metropolis-Coupled Markov Chain Monte Carlo (MCMCMC). The tree is midpoint rooted. Node labels represent posterior possibilities. The scale bar represents 0.2 amino acid substitutions per site. Different classes of NK genes are colour-coded. Species abbreviations: Ag, *Acanthoscurria geniculata* (Chelicerata); Dm, *Drosophila melanogaster* (Insecta); Ek, *Euperipatoides kanangrensis* (Onychophora); Gm, *Glomeris marginata* (Myriapoda); Pc, *Priapulus caudatus* (Priapulida); Pt, *Parasteatoda tepidariorum* (Chelicerata); Po, *Phalangium opilio* (Chelicerata). Additional protein abbreviations: Abox, Absent in olfactores; Bari, Bar-related in invertebrates homeobox; BarH, BarH-like homeobox; Barx; Bsx, Brain-specific homeobox; Dbx, developing brain homeobox; Dll, Distal-less; En, Engrailed; Evx, Even-skipped; Meox, Mesenchyme homeobox; Mnx, Motorneuron and pancreas homeobox; Nedex, Next to distal-less homeobox; Ro, rough; Vax, Ventral anterior homeobox.

Supplementary Material 3: Alignment CoreTree + *Priapulus caudatus*.

Supplementary Material 4: AllButChelTree. Bayesian analysis using MrBayes applying five million cycles for the Metropolis-Coupled Markov Chain Monte Carlo (MCMCMC). The tree is midpoint rooted. Node labels represent posterior possibilities. The scale bar represents 0.2 amino acid substitutions per site. Different classes of NK genes are colour-coded. Species abbreviations: Ag, *Acanthoscurria geniculata* (Chelicerata); Dm, *Drosophila melanogaster* (Insecta); Dp, *Daphnia pulex* (Branchiopoda); Ek, *Euperipatoides kanangrensis* (Onychophora); Gm, *Glomeris marginata* (Myriapoda); Pc, *Priapulus caudatus* (Priapulida); Pt, *Parasteatoda tepidariorum* (Chelicerata); Po, *Phalangium opilio* (Chelicerata); Rv, *Ramazzottius varieornatus* (Tardigrada); Sm, *Strigamia maritima* (Myriapoda); *Tribolium castaneum* (Insecta).

Supplementary Material 5: Alignment AllButChelTree.

Supplementary Material 6: ChelOnlyTree. Bayesian analysis using MrBayes applying four million cycles for the Metropolis-Coupled Markov Chain Monte Carlo (MCMCMC). The tree is midpoint rooted. Node labels represent posterior possibilities. The scale bar represents 0.2 amino acid substitutions per site. Different classes of NK genes are colour-coded. Species abbreviations: Cs, *Centruroides sculpturatus*; Ca, *Charinus acosta*; Hd, *Halotydeus destructor*; Mg, *Mastigoproctus giganteus*; Is, *Ixodes scapularis*; Pt, *Parasteatoda tepidariorum*; Pl, *Pycnogonum litorale*; Po, *Phalangium opilio*.

Supplementary Material 7: Alignment ChelOnlyTree.

Supplementary Material 8: MyriapodTree. Bayesian analysis using MrBayes applying two million cycles for the Metropolis-Coupled Markov Chain Monte Carlo (MCMCMC). The tree is midpoint rooted. Node labels represent posterior possibilities. The scale bar represents 0.2 amino acid substitutions per site. Different classes of NK genes are colour-coded. Species abbreviations: Dm, *Drosophila melanogaster*; Gm, *Glomeris marginata*; Hh, *Helicorthomorpha holstii*; Sm, *Strigamia maritima*; Tco, *Trigoniulus corallinus*.

Supplementary Material 9: Alignment MyriapodTree. Homeodomain sequences of *Trigoniulus corallinus* (Tco) and *Helicorthomorpha holstii* (Hh) NK genes are taken from Ou et al. (2020).

Supplementary Material 10: Primer Sequences.

Supplementary Material 11: Extended Complement. Each box represents one gene ortholog (or paralogs (staggered bars)). Different orthologs are colour-coded. The “X” symbol stands for putative absence of the gene. The grey shade marks species representing Arachnopulmonata for which a whole genome duplication was suggested; note the high number of duplicated genes in these species. Genes are subdivided into the core genes of the cluster NK1 (and associated genes), and genes of cluster NK2. Schematic drawings of the species are taken from PhyloPic (https://www.phylopic.org). Full species names: *Drosophila melanogaster*, *Tribolium castaneum*, *Daphnia pulex*, *Glomeris marginata*, *Trigoniulus corallinus*,* Strigamia maritima*, *Pycnogonum litorale*, *Phalangium opilio*, *Ixodes scapularis*, *Halotydeus destructor*, *Centruroides sculpturatus*, *Mastigoproctus giganteus*, *Charinus acosta*, *Parasteatoda tepidariorum*, *Euperipatoides kanangrensis*, *Ramazzottius varieornatus*, *Priapulus caudatus*.

Supplementary Material 12

## Data Availability

All data generated or analyzed during this study are included in this published article and its supplementary information files.
