# Supplementary figures and images for: Conserved and diverged embryonic expression patterns of panarthropod NK-Cluster genes and new evidence for CRE-shuffling
Source: BMC Ecol Evol. 2026 Apr 3;26:36. doi: 10.1186/s12862-026-02513-z (PMC13088399; doi:10.1186/s12862-026-02513-z)

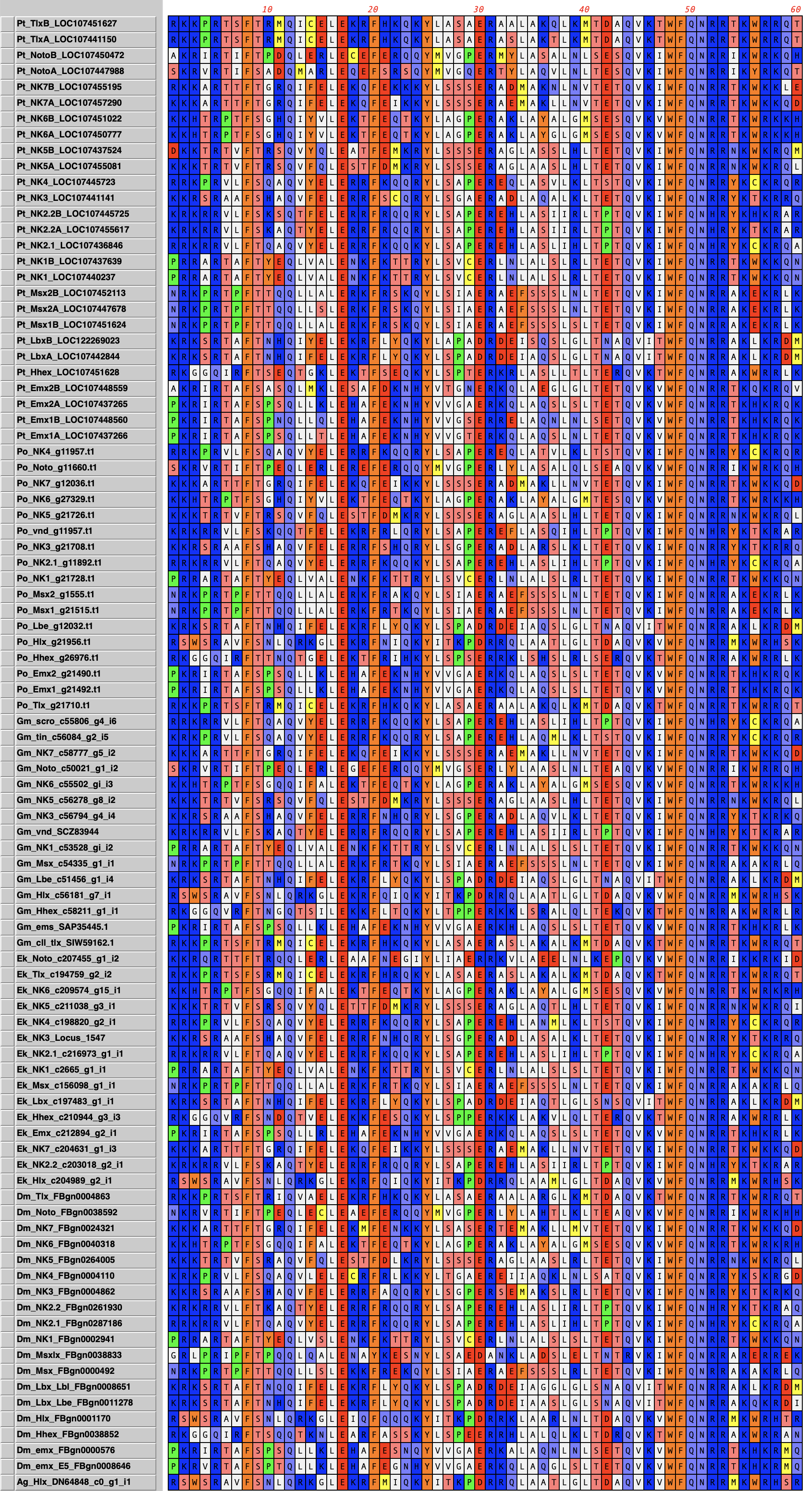

Supplement: Supplementary file 1 — Supplementary Material 1: Alignment CoreTree. [file 12862_2026_2513_MOESM1_ESM.tif]

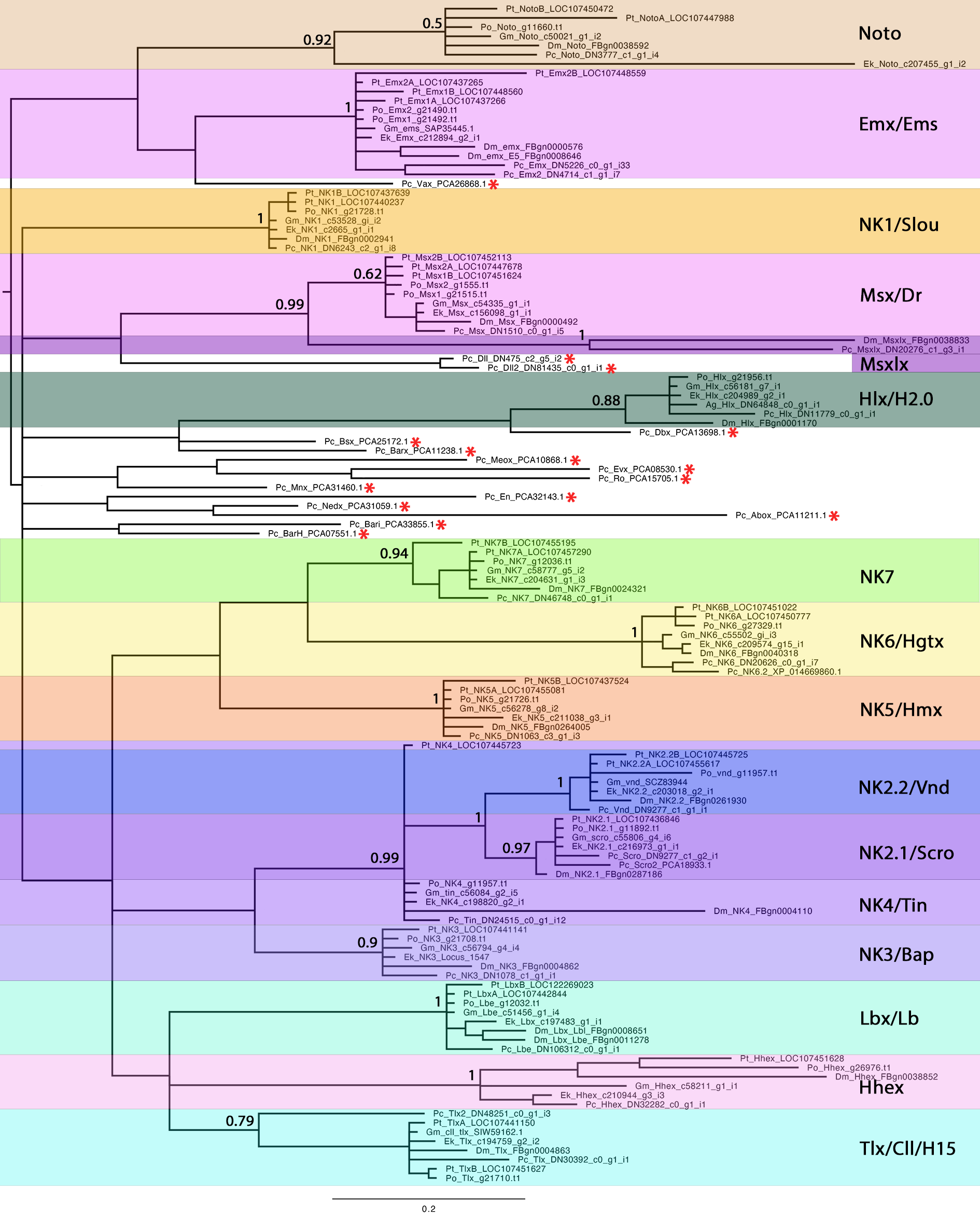

Supplement: Supplementary file 2 — Supplementary Material 2: CoreTree + Priapulus caudatus. Bayesian analysis using MrBayes applying three million cycles for the Metropolis-Coupled Markov Chain Monte Carlo (MCMCMC). The tree is midpoint rooted. Node labels represent posterior possibilities. The scale bar represents 0.2 amino acid substitutions per site. Different classes of NK genes are colour-coded. Species abbreviations: Ag, Acanthoscurria geniculata (Chelicerata); Dm, Drosophila melanogaster (Insecta); Ek, Euperipatoides kanangrensis (Onychophora); Gm, Glomeris marginata (Myriapoda); Pc, Priapulus caudatus (Priapulida); Pt, Parasteatoda tepidariorum (Chelicerata); Po, Phalangium opilio (Chelicerata). Additional protein abbreviations: Abox, Absent in olfactores; Bari, Bar-related in invertebrates homeobox; BarH, BarH-like homeobox; Barx; Bsx, Brain-specific homeobox; Dbx, developing brain homeobox; Dll, Distal-less; En, Engrailed; Evx, Even-skipped; Meox, Mesenchyme homeobox; Mnx, Motorneuron and pancreas homeobox; Nedex, Next to distal-less homeobox; Ro, rough; Vax, Ventral anterior homeobox. [file 12862_2026_2513_MOESM2_ESM.tif]

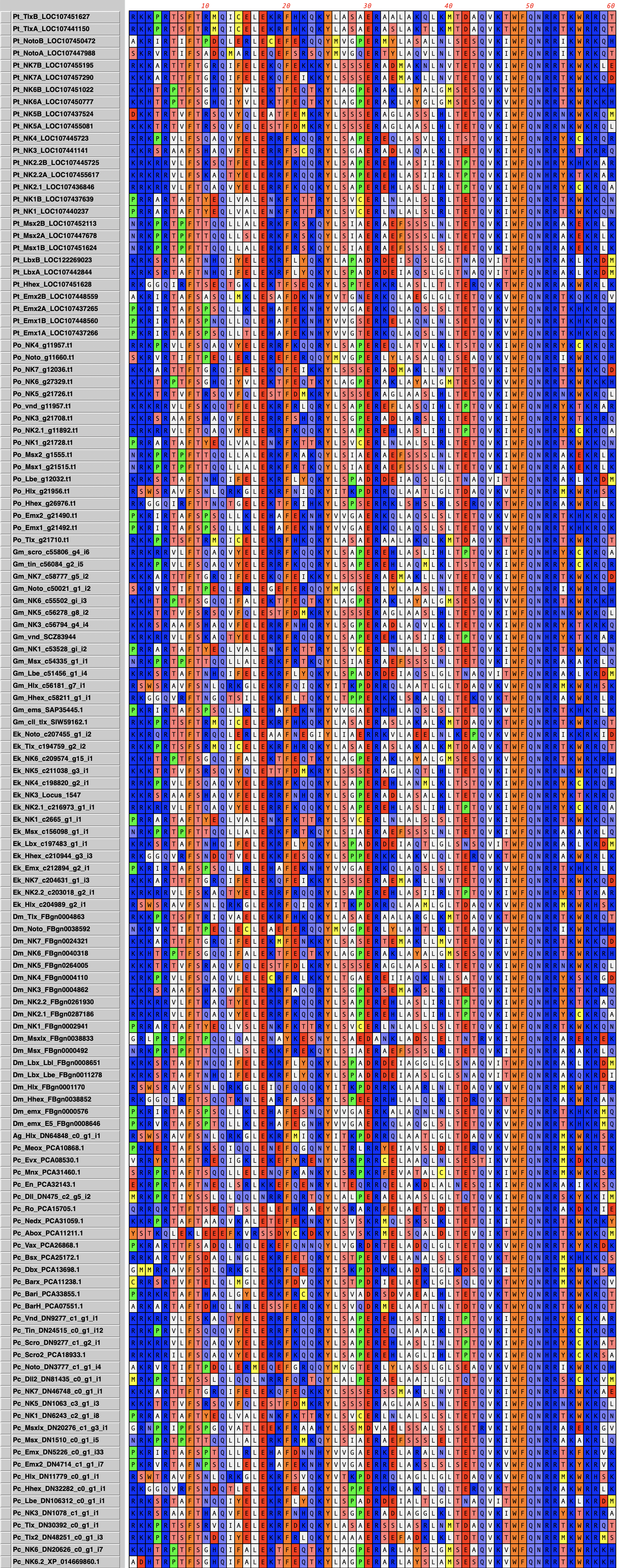

Supplement: Supplementary file 3 — Supplementary Material 3: Alignment CoreTree + Priapulus caudatus. [file 12862_2026_2513_MOESM3_ESM.tif]

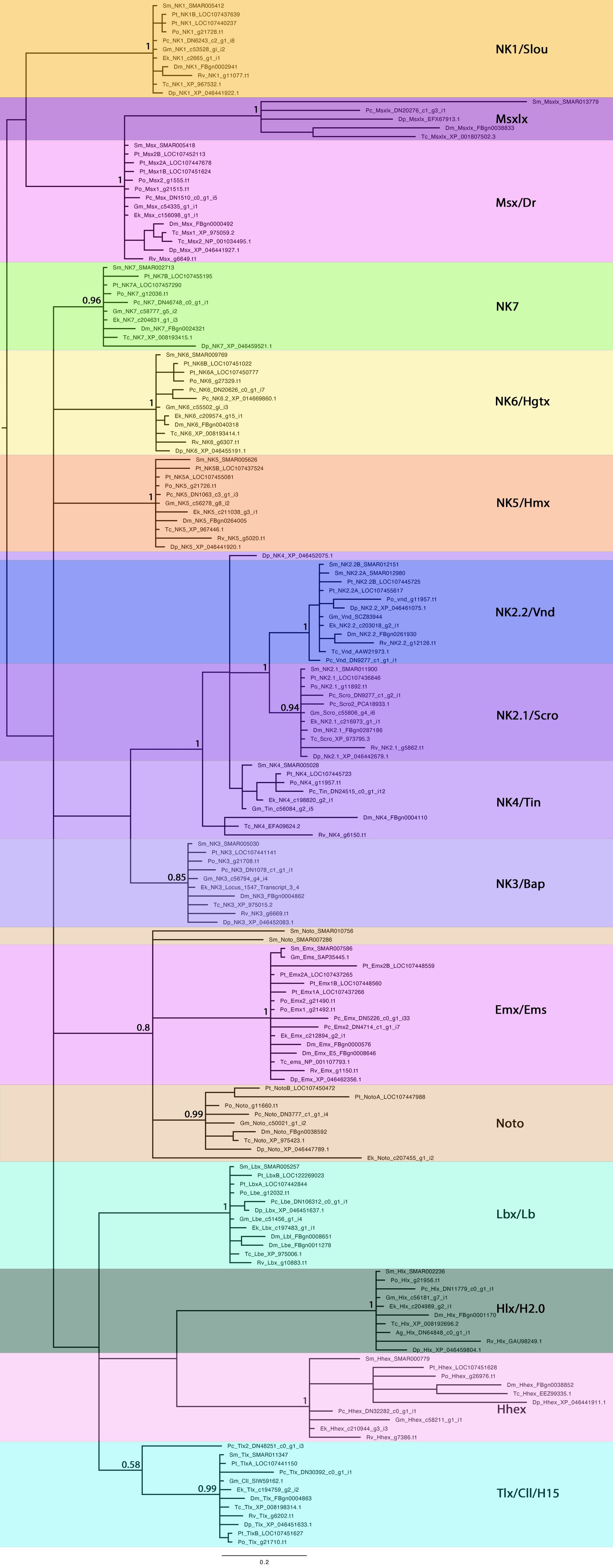

Supplement: Supplementary file 4 — Supplementary Material 4: AllButChelTree. Bayesian analysis using MrBayes applying five million cycles for the Metropolis-Coupled Markov Chain Monte Carlo (MCMCMC). The tree is midpoint rooted. Node labels represent posterior possibilities. The scale bar represents 0.2 amino acid substitutions per site. Different classes of NK genes are colour-coded. Species abbreviations: Ag, Acanthoscurria geniculata (Chelicerata); Dm, Drosophila melanogaster (Insecta); Dp, Daphnia pulex (Branchiopoda); Ek, Euperipatoides kanangrensis (Onychophora); Gm, Glomeris marginata (Myriapoda); Pc, Priapulus caudatus (Priapulida); Pt, Parasteatoda tepidariorum (Chelicerata); Po, Phalangium opilio (Chelicerata); Rv, Ramazzottius varieornatus (Tardigrada); Sm, Strigamia maritima (Myriapoda); Tribolium castaneum (Insecta). [file 12862_2026_2513_MOESM4_ESM.tif]

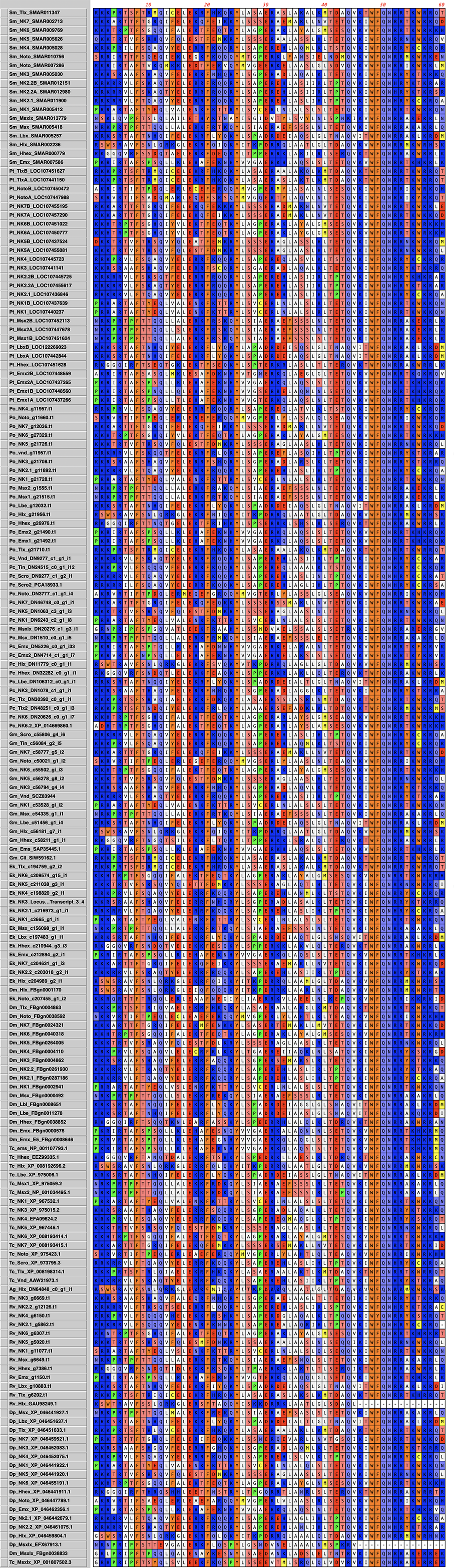

Supplement: Supplementary file 5 — Supplementary Material 5: Alignment AllButChelTree. [file 12862_2026_2513_MOESM5_ESM.tif]

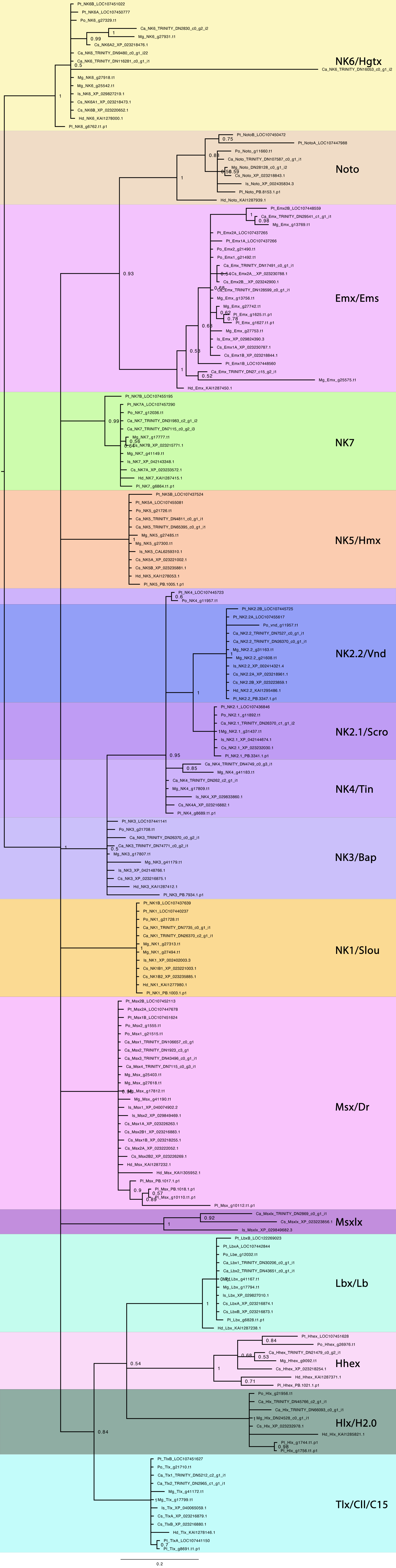

Supplement: Supplementary file 6 — Supplementary Material 6: ChelOnlyTree. Bayesian analysis using MrBayes applying four million cycles for the Metropolis-Coupled Markov Chain Monte Carlo (MCMCMC). The tree is midpoint rooted. Node labels represent posterior possibilities. The scale bar represents 0.2 amino acid substitutions per site. Different classes of NK genes are colour-coded. Species abbreviations: Cs, Centruroides sculpturatus; Ca, Charinus acosta; Hd, Halotydeus destructor; Mg, Mastigoproctus giganteus; Is, Ixodes scapularis; Pt, Parasteatoda tepidariorum; Pl, Pycnogonum litorale; Po, Phalangium opilio. [file 12862_2026_2513_MOESM6_ESM.tif]

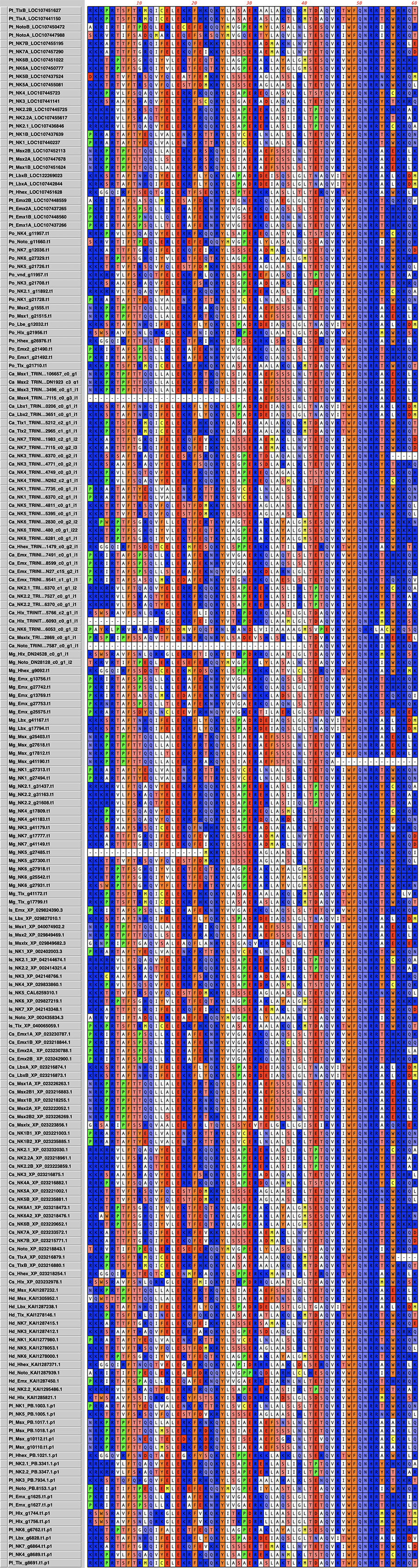

Supplement: Supplementary file 7 — Supplementary Material 7: Alignment ChelOnlyTree. [file 12862_2026_2513_MOESM7_ESM.tif]

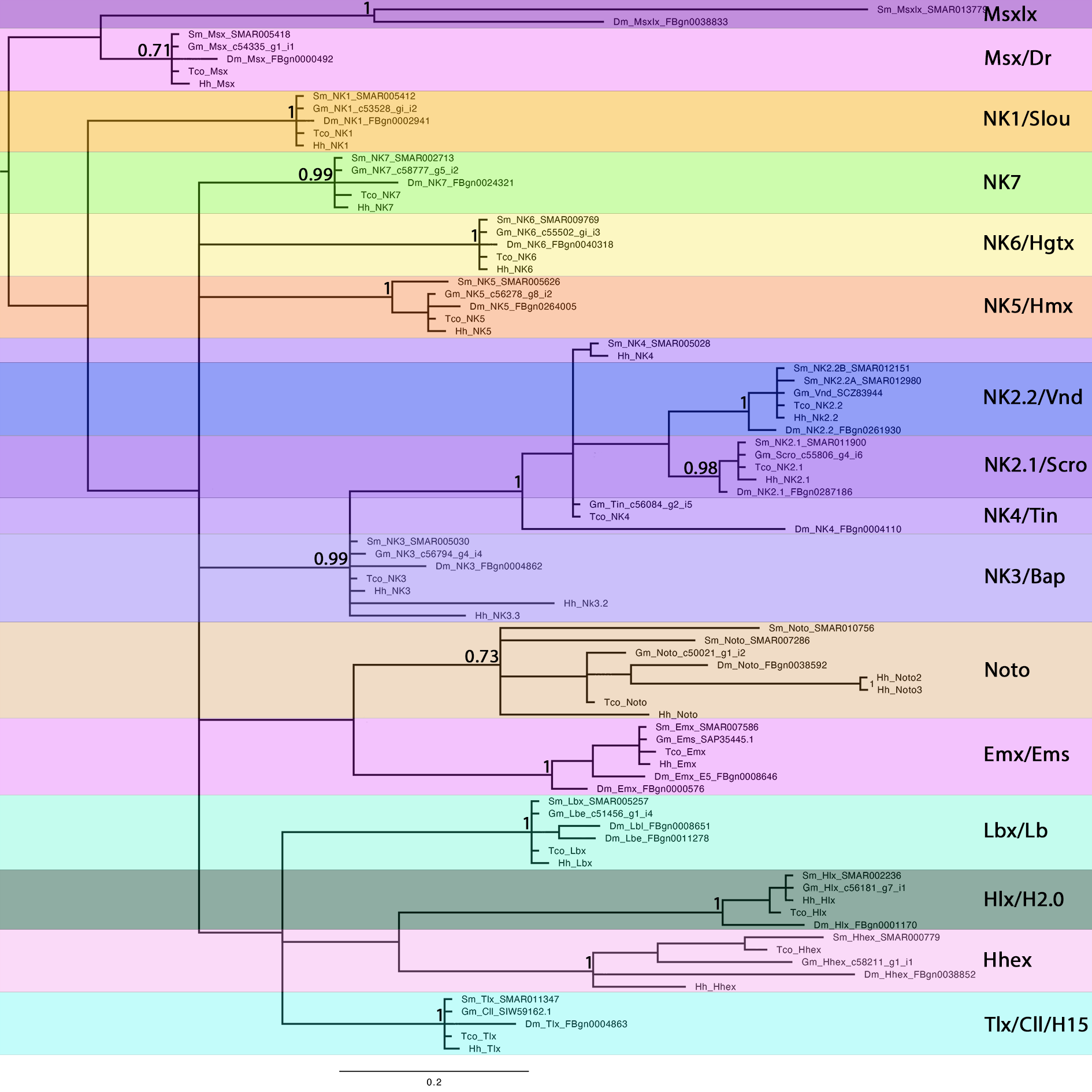

Supplement: Supplementary file 8 — Supplementary Material 8: MyriapodTree. Bayesian analysis using MrBayes applying two million cycles for the Metropolis-Coupled Markov Chain Monte Carlo (MCMCMC). The tree is midpoint rooted. Node labels represent posterior possibilities. The scale bar represents 0.2 amino acid substitutions per site. Different classes of NK genes are colour-coded. Species abbreviations: Dm, Drosophila melanogaster; Gm, Glomeris marginata; Hh, Helicorthomorpha holstii; Sm, Strigamia maritima; Tco, Trigoniulus corallinus. [file 12862_2026_2513_MOESM8_ESM.tif]

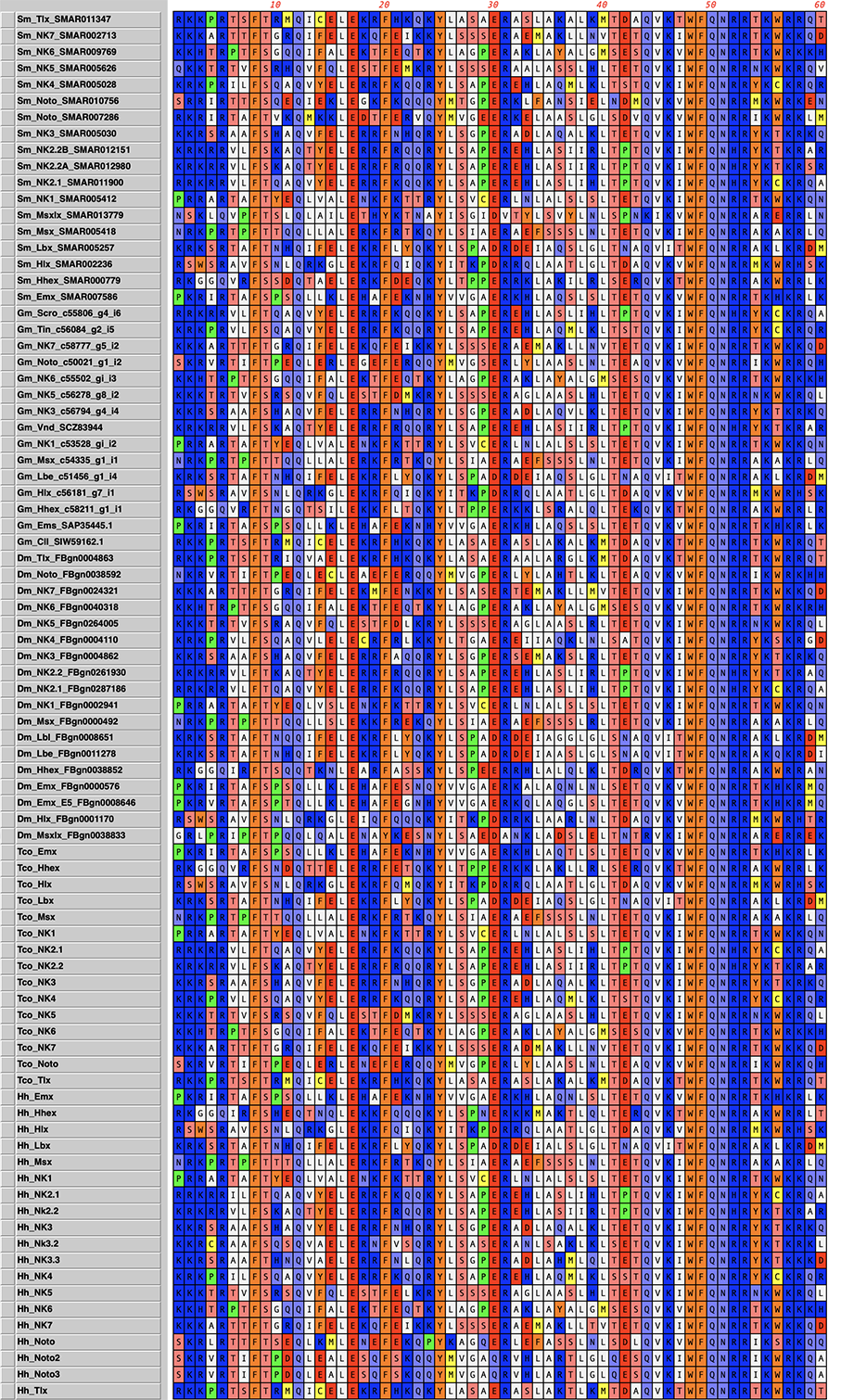

Supplement: Supplementary file 9 — Supplementary Material 9: Alignment MyriapodTree. Homeodomain sequences of Trigoniulus corallinus (Tco) and Helicorthomorpha holstii (Hh) NK genes are taken from Ou et al. (2020). [file 12862_2026_2513_MOESM9_ESM.tif]

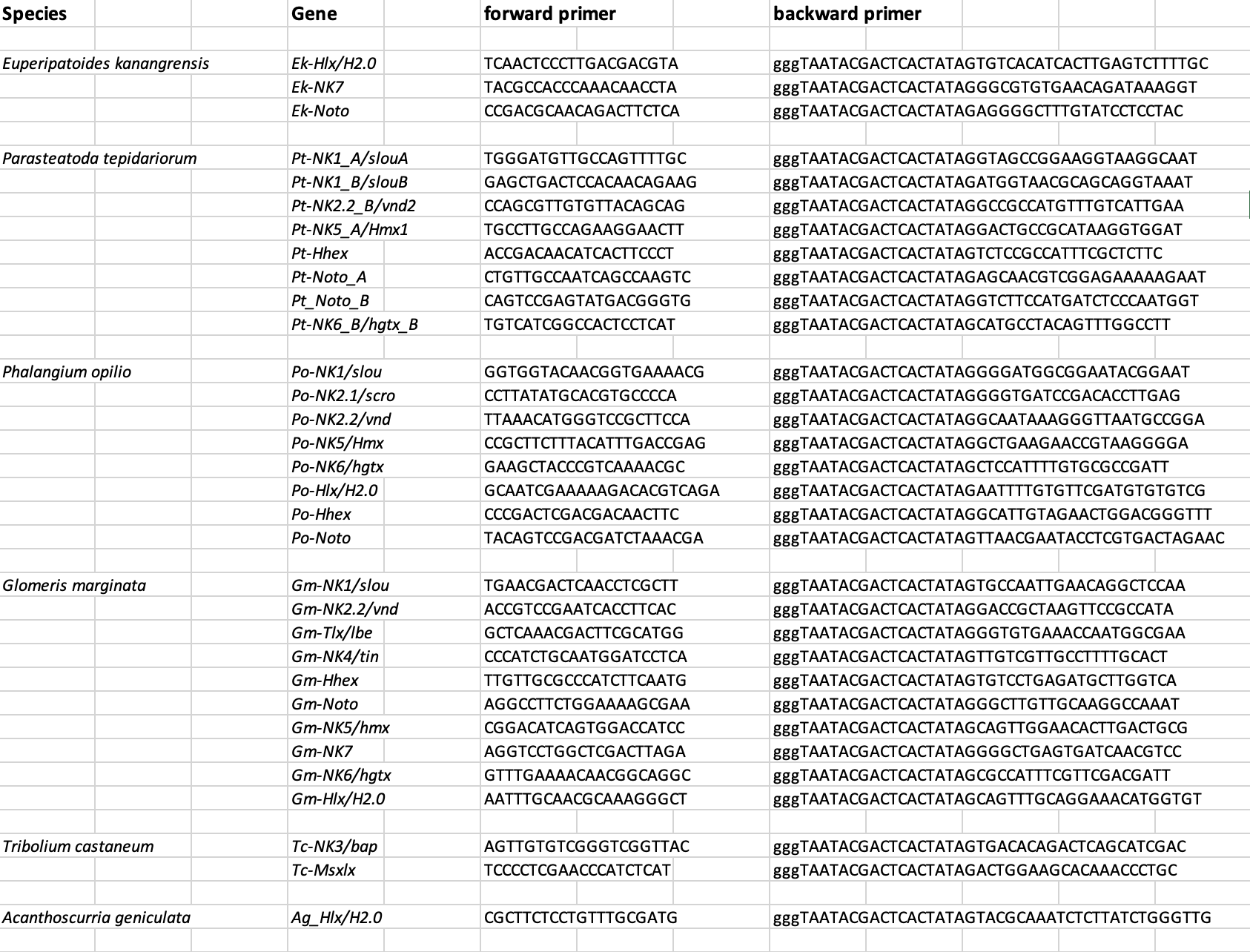

Supplement: Supplementary file 10 — Supplementary Material 10: Primer Sequences. [file 12862_2026_2513_MOESM10_ESM.tif]

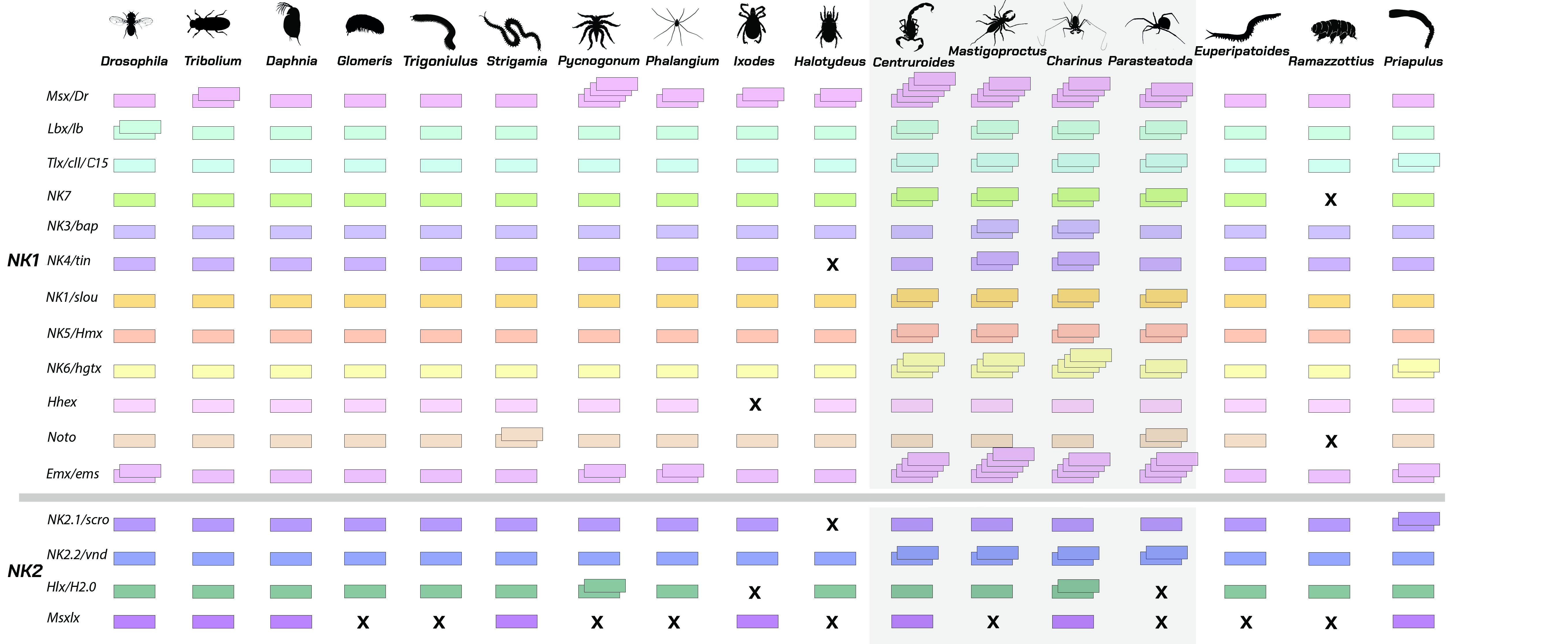

Supplement: Supplementary file 12 — Supplementary Material 12 [file 12862_2026_2513_MOESM12_ESM.zip › 12862_2026_2513_MOESM12_ESM.tif]
